# Designing spin and orbital sources of Berry curvature at oxide interfaces

**DOI:** 10.1038/s41563-023-01498-0

**Published:** 2023-03-16

**Authors:** Edouard Lesne, Yildiz G. Saǧlam, Raffaele Battilomo, Maria Teresa Mercaldo, Thierry C. van Thiel, Ulderico Filippozzi, Canio Noce, Mario Cuoco, Gary A. Steele, Carmine Ortix, Andrea D. Caviglia

**Affiliations:** 1 Kavli Institute of Nanoscience, Delft University of Technology, Delft, the Netherlands; 2 Institute for Theoretical Physics, Center for Extreme Matter and Emergent Phenomena, Utrecht University, Utrecht, the Netherlands; 3 Dipartimento di Fisica ‘E. R. Caianiello’, Universitá di Salerno, Fisciano, Italy; 4 CNR-SPIN c/o Universita’ di Salerno, Fisciano, Italy; 5 Department of Quantum Matter Physics, University of Geneva, Geneva, Switzerland; 6 Present Address: Max Planck Institute for Chemical Physics of Solids, Dresden, Germany

**Keywords:** Electronic properties and materials, Surfaces, interfaces and thin films

## Abstract

Quantum materials can display physical phenomena rooted in the geometry of electronic wavefunctions. The corresponding geometric tensor is characterized by an emergent field known as the Berry curvature (BC). Large BCs typically arise when electronic states with different spin, orbital or sublattice quantum numbers hybridize at finite crystal momentum. In all the materials known to date, the BC is triggered by the hybridization of a single type of quantum number. Here we report the discovery of the first material system having both spin- and orbital-sourced BC: LaAlO_3_/SrTiO_3_ interfaces grown along the [111] direction. We independently detect these two sources and probe the BC associated to the spin quantum number through the measurements of an anomalous planar Hall effect. The observation of a nonlinear Hall effect with time-reversal symmetry signals large orbital-mediated BC dipoles. The coexistence of different forms of BC enables the combination of spintronic and optoelectronic functionalities in a single material.

## Abstract (web summary)

We report both spin- and orbital-sourced Berry curvature in LaAlO_3_/SrTiO_3_ interfaces grown along the [111] direction.

## Main

When moving along closed paths, electrons can accumulate a geometric Berry phase related to the flux of a field, called the Berry curvature (BC), encoding the geometric properties of the electronic wavefunctions. In magnetic materials, the adiabatic motion of electrons around the Fermi surface provides such a Berry phase. It is directly observable since it governs the intrinsic part of the anomalous Hall conductivity^1,2^. Anomalous Hall effect measurements, therefore, represent a charge transport footprint of the intrinsic geometric structure of electronic wavefunctions. In non-magnetic materials, the BC field is forced to vanish by symmetry when summed over the occupied electronic states. However, local concentrations of positive and negative BC in momentum space are allowed by acentric crystalline arrangements^3^. This segregation of BC in different regions of momentum space appears whenever electronic states with different internal quantum numbers are coupled to each other by terms that linearly depend on crystalline momentum *k*. In these regions, the electronic bands typically resemble the dispersion relations of relativistic Dirac or Weyl fermions. The spin–orbit linear-in-*k* coupling between different spin states shapes the Dirac cones at the surfaces of three-dimensional topological insulators^4,5^ as well as the Weyl cones of topological semimetals^6^. Couplings between different atomic orbital and sublattice states, instead, give rise to the (gapped) Dirac cones of transitional metal dichalcogenides and graphene. Conceptually speaking, the appearance of BC beyond this Dirac/Weyl paradigm is entirely allowed. The fundamental conditions for the occurrence of BC only involve the crystalline geometry of a material, with no restrictions on the specific properties of its low-energy electronic excitations. Achieving this challenge is of great interest. First, it could, in principle, result in the coexistence of different mechanisms of BC generation. This could be used, in turn, to endow a single-material system with different BC-mediated effects, for instance, spin and orbital Hall effects. Second, searching for BCs without Dirac or Weyl cones might allow the design of materials with interplay of correlated and topological physics—an unexplored frontier in condensed-matter physics.

Here we reach these two milestones in the two-dimensional electron system (2DES) confined at (111)-oriented oxide interfaces, with a high-temperature trigonal crystalline structure. This model system satisfies the crystalline symmetry properties for a non-vanishing BC. The combination of spin–orbit coupling, orbital degrees of freedom associated with the low-energy *t*_2g_ electrons, and crystal fields leads to the coexistence of a spin-sourced and orbital-sourced BC. The two sources are independently probed using two different charge transport diagnostic tools. The observation of the BC-mediated anomalous planar Hall effect (APHE)^7,8^ grants direct access to the spin-sourced BC, whereas nonlinear Hall transport measurements in time-reversal symmetric conditions^9,10^ detect an orbital-mediated Berry curvature dipole (BCD)—a quantity measured so far only in gapped Dirac systems^9–19^ and three-dimensional topological semimetals^20–25^. We identify (111)-oriented LaAlO_3_/SrTiO_3_ heterointerfaces as an ideal material system because their 2DES features many-body correlations and a two-dimensional superconducting ground state^26–30^.

**Fig. 1:**
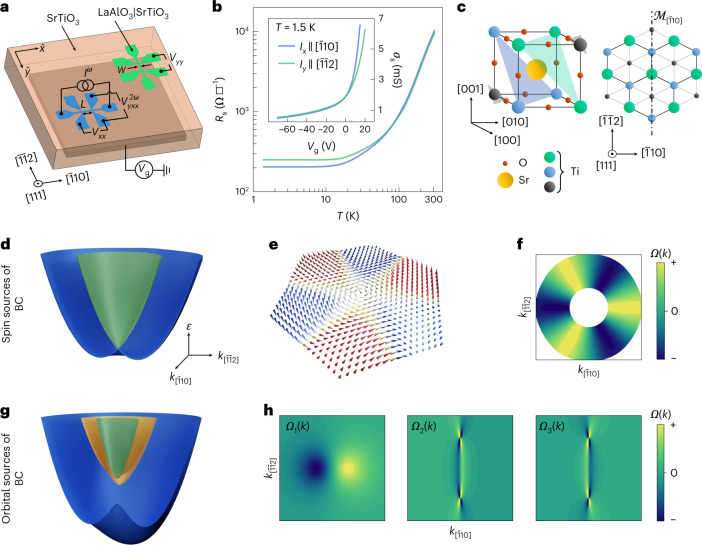
Crystal and model band structures of the (111)-oriented LaAlO_3_/SrTiO_3_ 2DES and basic electrical characterization. **a**, Schematic of the electrical measurement configurations of two Hall bars, hosting a 2DES, and oriented along the $[\bar{1}10]$ and $[\bar{1}\bar{1}2]$ crystallographic axes. Here *W* is the width of the channel and *L* is the distance between the longitudinal voltage probes. Also, *V*_g_ stands for the high-voltage source used to tune the 2DES band occupation (Fermi energy) in a back-gate geometry. **b**, Sheet resistance *R*_s_ versus temperature *T* of the 2DES for the $[\bar{1}10]$ and $[\bar{1}\bar{1}2]$ Hall-bar devices, showing a nearly isotropic metallic character. The inset shows the sheet conductance, ${\sigma }_{{{{\rm{s}}}}}={R}_{{{{\rm{s}}}}}^{-1}$, as a function of back-gate voltage *V*_g_, showing gate tunability of the 2DES at 1.5 K. **c**, Schematic of SrTiO_3_ perovskite cubic unit cell displaying the non-equivalent (111) titanium planes (shaded areas) (left). Top view along the [111] crystallographic direction, of the same unit cell, showing only the Ti atoms (right). The dash–dotted line indicates the mirror line ${{{{\mathcal{M}}}}}_{[\bar{1}10]}$. **d**, Schematic of a single pair of spin-split bands forming a Kramers’ pair at the Γ point up to the Fermi level. **e**, Each spin band is characterized by a non-trivial spin texture with out-of-plane spin components induced by the effect of trigonal warping. **f**, Exclusion plot of the BC *Ω*_*k*_ over the Fermi surfaces of the two spin sub-bands. **g**, Schematic of the band structure of spin–orbit-free orbital bands corresponding to *t*_2g_ electrons subject to a ${{{{\mathcal{C}}}}}_{\mathrm{s}}$ crystal field. At the centre of the BZ, all the levels are split. The orbital Rashba coupling (∝*α*_m_) leads to mirror-symmetry-protected crossings. **h**, Band-resolved BC displaying dipolar hotspots (left) in the lowest-energy band and singular pinch points in the highest-energy bands.

We synthesize (111)-oriented LaAlO_3_/SrTiO_3_ heterostructures by pulsed laser deposition (Methods). The samples are lithographically patterned into Hall bars oriented along the two orthogonal principal in-plane crystallographic directions: the $[\bar{1}10]$ and $[\bar{1}\bar{1}2]$ axis (Fig. 1a). The sheet conductance and carrier density of the 2DES are controlled by electrostatic-field effects in a back-gate geometry (Fig. 1b). We source an oscillating current (*I*^*ω*^) with frequency *ω*/2π along each Hall bar, and concomitantly measure the longitudinal response as well as the first- or second-harmonic transverse voltages in a conventional lock-in detection scheme (Fig. 1a).

The non-trivial geometric properties of the electronic waves in the 2DES derive entirely from the triangular arrangement of the titanium atoms at the (111)-oriented LaAlO_3_/SrTiO_3_ interface (Fig. 1c). Together with the ${{{{\mathcal{M}}}}}_{\bar{1}10}$ mirror-line symmetry, this yields a ${{{{\mathcal{C}}}}}_{3v}$ crystallographic point group symmetry. As a result of this trigonal crystal field and the concomitant presence of spin–orbit coupling, the entire *d*-orbital manifold of Ti atoms located at the centre of the surface Brillouin zone (BZ) is split into five distinct Kramers’ pairs (Supplementary Note I). The energy bands of the pairs are shifted in momentum due to spin–orbit coupling. In their simplest form, they acquire a parabolic dispersion reminiscent of a Rashba 2DES (Fig. 1d). However, the trigonal crystal field brings about a specific hexagonal warping^31,32^ that has a twofold effect. First, for each time-reversal related pair of bands, the Fermi lines acquire a hexagonal ‘snowflake’ shape^33^. Second, and the most important, the spin texture in momentum space acquires a characteristic out-of-plane component^34,35^, with alternating meron and antimeron wedges respecting the symmetry properties of the crystal (Fig. 1e). This unique spin–momentum locking enables a non-vanishing local BC entirely generated by spin–orbit coupling (Supplementary Note I). The local BC of the spin-split bands of each pair cancel each other at the same crystal momentum. However, there is a region of crystal momenta populated by a single spin band. In this region (namely, the annulus between the two Fermi lines of the system), alternating positive and negative regions of non-vanishing BC are present (Fig. 1f).

Apart from the spin channel, an inherently different source of BC exists. In systems with orbital degrees of freedom, the lack of crystal centrosymmetry yields coupling that are linear in *k*, and mix different atomic orbital states. These orbital Rashba couplings^36^ are independent of the presence of spin–orbit coupling. Precisely as its spin counterpart, the orbital Rashba coupling can generate a finite BC^37^, but only when all the rotational symmetries are broken (Methods and Supplementary Note I). With a reduced ${{{{\mathcal{C}}}}}_{\mathrm{s}}$ symmetry, low-lying *t*_2g_ orbitals are split into three non-degenerate levels. The corresponding orbital bands then realize a gapped Rashba-like spectrum with protected crossings along the mirror-symmetric lines of the two-dimensional BZ (Fig. 1g). These characteristics result in the appearance of dipolar BC hotspots and singular pinch points (Fig. 1h). Such orbital sources of BC are fully active at the (111) oxide interfaces owing to the reduced low-temperature symmetries. The cubic-to-tetragonal structural phase transition^38,39^ occurring at 110 K breaks the three-fold rotational symmetry along the [111] direction. In addition, the tetragonal to locally triclinic structural distortions at temperatures below ~70 K together with the ferroelectric instability^40^ below 50 K are expected to strongly enhance the orbital Rashba strength.

**Fig. 2:**
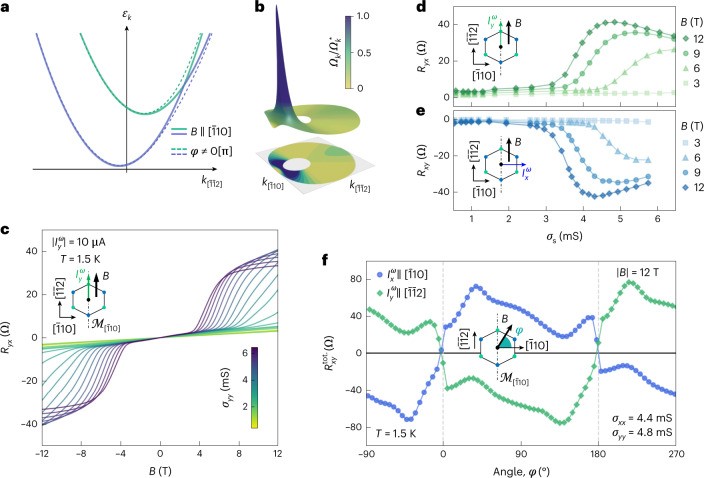
APHE response of the 2DES induced by the spin-sourced BC. **a**, Schematic of the energy dispersion of the spin-split bands along the mirror line of the BZ ${k}_{[\bar{1}10]}=0$ in the presence of a planar magnetic field. When the latter is oriented along the $[\bar{1}10]$ direction, there is a mirror-symmetry-protected crossing of the spin-split bands that evolves into an anticrossing for the other directions of the magnetic field. Angle *φ* is defined by the orientation of the magnetic field with respect to the $[\bar{1}10]$ crystallographic direction (schematic shown in the inset of **f**). **b**, Sketch of the spin-sourced BC-normalized magnitude ${{{\varOmega }}}_{k}/{{{\varOmega }}}_{k}^{\star }$ when the magnetic field is directed along the $[\bar{1}\bar{1}2]$ direction. When the anticrossing point enters the Fermi surface annulus, the integral of the BC is strongly enhanced and the APHE response reaches its maximum. **c**, Experimentally measured field-antisymmetric planar Hall resistance ${R}_{{{{{xy}}}}}={V}_{{{{{xy}}}}}^{\omega }/{I}_{{{{{x}}}}}^{\omega }$ at *T* = 1.5 K, with ${I}_{{{{{y}}}}}^{\omega }$ along $[\bar{1}\bar{1}2]\parallel B$ (schematic in the inset), for different sheet conductance values *σ*_*yy*_ indicated by the coloured scale bar. **d**, Corresponding dependence of *R*_*yx*_ versus *σ*_*yy*_ showing a non-monotonic behaviour for fixed values of *B* > 3 T, and an onset above a threshold value of *σ*_*yy*_. **e**, Dependence of the field-antisymmetric contribution *R*_*xy*_ versus *σ*_*xx*_ for ${I}_{{{{{x}}}}}^{\omega }$ along $[\bar{1}10]\perp B$ (Extended Data Fig. 1). **f**, In-plane angular dependence of the raw total transverse-resistance response ${R}_{{{{{xy}}}}}^{{{{\rm{tot.}}}}}$, that is, not field-(anti)symmetrized, for the two Hall-bar devices at *B* = 12 T. The planar Hall response obeys the Onsager relation *R*_*xy*_(*B*) = *R*_yx_(–*B*), as evident from the near-identical angular dependence on imposing a ±π translation to either curve. Remarkably, ${R}_{{{{{xy}}}}}^{{{{\rm{tot.}}}}}$ goes to zero at *φ* = 0° and *φ* = 180°, that is, when the mirror symmetry is preserved even in the presence of an external magnetic field.

The orbital-sourced BC is expected to be very stiff in response to externally applied in-plane magnetic fields due to the absence of symmetry-protected orbital degeneracies. In contrast, the spin-sourced BC is substantially more susceptible to planar magnetic fields. As shown in Fig. 2a,b, an in-plane magnetic field is capable of generating a BC hotspot within the Fermi surface annulus. This BC hotspot corresponds to a field-induced avoided level crossing between the two spin-split bands that occurs whenever the applied magnetic field breaks the residual crystalline mirror symmetry. The momentum-integrated net BC is then non-zero (Supplementary Note II), and yields a transverse Hall conductance satisfying the antisymmetric property *σ*_*xy*_*ρ*_*yx*_ = −1, even in the absence of any Lorentz force. This effect, theoretically predicted elsewhere^7,8^ and known as the APHE, is different in nature with respect to the conventional planar Hall effect, which is instead related to the anisotropy in the longitudinal magnetoresistance and thus characterized by a symmetric response, namely, *σ*_*xy*_(*B*) = *σ*_*xy*_(–*B*).

**Extended Data Fig. 3:**
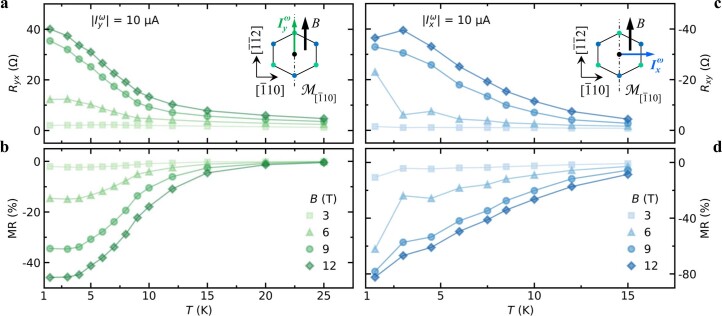
Temperature dependence of the Hall and longitudinal magnetoresistances in a planar magnetic field. Temperature dependent planar Hall resistance (**a**,**c**) and longitudinal MR (**b**,**d**) in the linear response regime for various strength of the in-plane magnetic field (and at fixed gate-voltage), with ${I}_{{{{\rm{y}}}}}^{\omega }$ along $[\bar{1}\bar{1}2]\parallel B$ (**a-b**) and ${I}_{{{{\rm{x}}}}}^{\omega }$ along $[\bar{1}10]\perp B$ (**c-d**). Both *R*_yx_ and *R*_xy_, as well as the corresponding longitudinal MR, asymptotically go to zero as the temperature increases toward strontium titanate’s nonpolar tetragonal phase (above ≈ 30 K).

**Extended Data Fig. 4:**
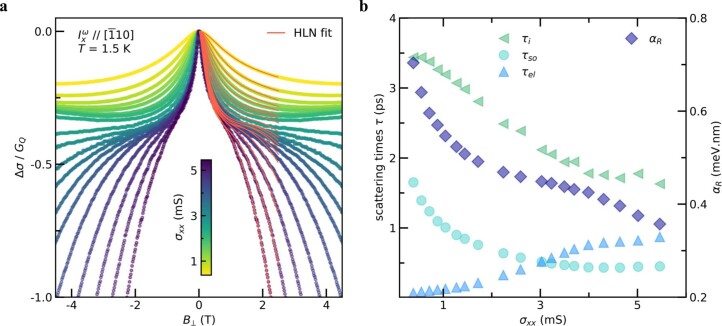
Weak antilocalization regime and Rashba spin-orbit coupling. **a**, Gate-modulated magnetoconductance curves (normalized to the quantum of conductance G_Q_) for ${I}_{{{{\rm{x}}}}}^{\omega }$ along $[\bar{1}10]$ (see also Supplementary Note III for WAL measurements along $[\bar{1}\bar{1}2]$). Hikami-Larkin-Nagaoka fits (solid red lines) are performed following Eq. (3). *B*_⊥_, the out-of-plane magnetic field. **(c)** Left axis: Experimentally estimated momentum, inelastic and spin-orbit relaxation times, *τ*_el_ (named *τ* throughout the manuscript), *τ*_i_ and *τ*_so_, respectively. Right axis: Strength of the Rashba spin-orbit coupling *α*_R_ versus sheet conductance *σ*_xx_.

**Extended Data Fig. 5:**
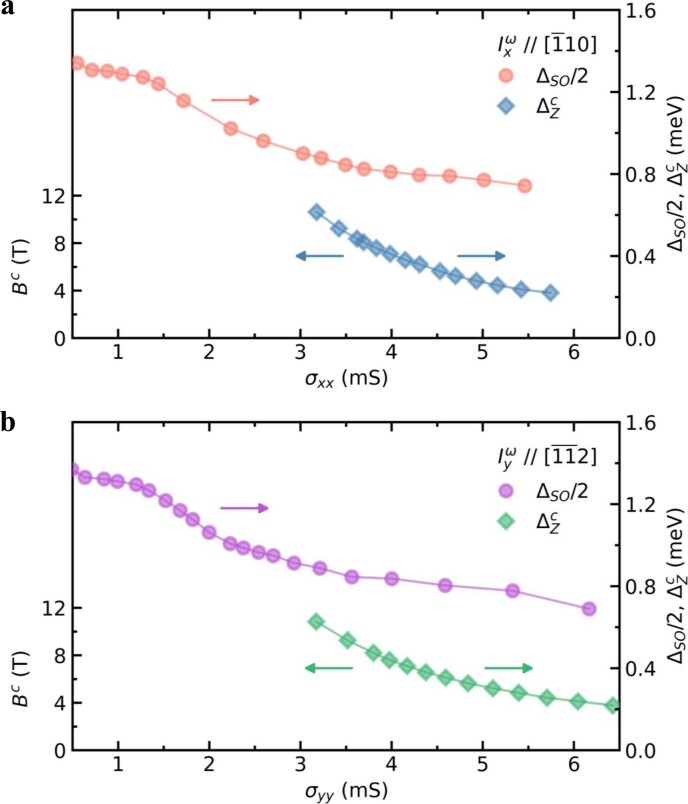
Effective Zeeman energy and Rashba spin-orbit energy of the 2DES. **a**-**b**, Right axis: Sheet conductance dependence of the Rashba spin-orbit energy Δ_so_/2 = *α*_R_*k*_F_ and effective Zeeman energy ${\Delta }_{{{{\rm{Z}}}}}^{{{{\rm{c}}}}}=g{\mu }_{{{{\rm{B}}}}}{B}^{{{{\rm{c}}}}}/2$ at the critical in-plane field *B*^c^ (left axis), for ${I}_{{{{\rm{x}}}}}^{\omega }$ along $[\bar{1}10]$ (panel **a**) and for ${I}_{{{{\rm{y}}}}}^{\omega }$ along $[\bar{1}\bar{1}2]$ (panel **b**). See Supplementary Note III for details regarding the determination of *B*^c^.

Figure 2c shows the transverse (Hall) resistance measured with a current applied along the $[\bar{1}\bar{1}2]$ crystal direction and with collinear current and magnetic field. This ensures a vanishing symmetric planar Hall effect^7^. At fields well below 4 T, a small signal increasing linearly with the field strength is detected. This feature can be attributed to an out-of-plane misalignment of the magnetic field smaller than 1.5° (Supplementary Note III). Above a magnetic-field threshold instead, a large transverse Hall signal sharply emerges (Extended Data Fig. 3). At even larger fields, this response saturates. Electrostatic gating is found to decrease the magnetic-field threshold and promotes a non-monotonic evolution of the response amplitude (Fig. 2d,e). The experimental features of this Hall response can be captured by considering a single pair of spin-split bands coupled to the external field by the Zeeman interaction. In this picture, the sudden onset of the transverse response is associated with the appearance of the BC hotspot inside the Fermi surface annulus occurring at a critical magnetic-field strength (Supplementary Note II). Magnetoconductance measurements in the weak antilocalization regime (Extended Data Fig. 4) show that the onset of the transverse Hall signal precisely coincides with the appearance of the spin-sourced BC hotspot (Extended Data Fig. 5). The non-monotonic behaviour of the transverse response as a function of electrostatic gating and magnetic-field strength can also be ascribed to the BC origin of the Hall response. The angular dependence of the transverse resistance (Fig. 2f) indicates a vanishing transverse linear conductivity when the planar magnetic field is along the $[\bar{1}10]$ direction, due to mirror symmetry ${{{{\mathcal{M}}}}}_{[\bar{1}10]}$. This is independent of whether the driving current is along the $[\bar{1}10]$ or $[\bar{1}\bar{1}2]$ direction. Note that the two angular dependencies are related to each other by a 180° shift, in agreement with the Onsager reciprocity relations^41^.

**Fig. 3:**
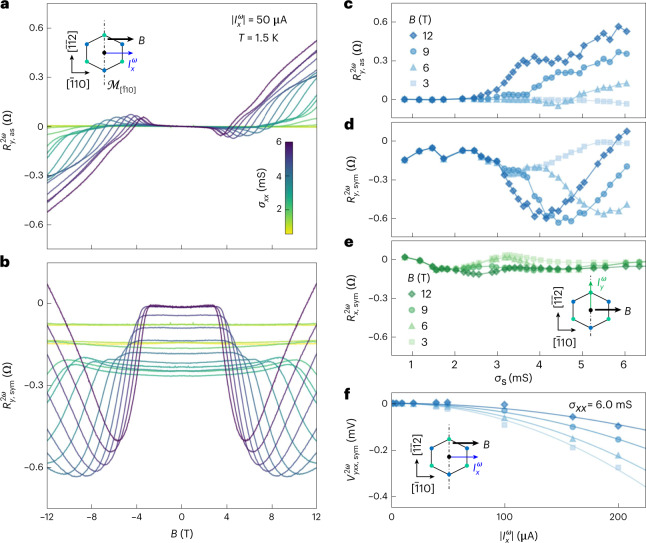
Nonlinear Hall response of the 2DES in a planar magnetic field. **a**,**b**, Field-antisymmetric ${R}_{{{{{y}}}},{{{\rm{as}}}}}^{2\omega }$ (**a**) and field-symmetric ${R}_{{{{{y}}}},{{{\rm{sym}}}}}^{2\omega }$ (**b**) second-harmonic transverse-resistance responses for ${I}_{{{{{x}}}}}^{\omega }$ along $[\bar{1}10]\parallel B$, and for different values of sheet conductance *σ*_*xx*_. **c**,**d**, Corresponding dependence of ${R}_{{{{{y}}}},{{{\rm{as}}}}}^{2\omega }$ (**c**) and ${R}_{{{{{y,\mathrm{sym}}}}}}^{2\omega }$ (**d**) versus *σ*_*xx*_ for different values of in-plane magnetic field *B*. The field-symmetric nonlinear transverse resistance displays a strong non-monotonic response, attributed to a Zeeman-induced Berry-phase contribution. **e**, Field-symmetric second-harmonic transverse-resistance response ${R}_{{{{{x}}}},{{{\rm{sym}}}}}^{2\omega }$ for ${I}_{{{{{y}}}}}^{\omega }$ along $[\bar{1}\bar{1}2]\perp B$ versus sheet conductance *σ*_*yy*_ at fixed values of the planar magnetic field (Extended Data Fig. 2). The full-scale ordinate axis is chosen to be the same as that in **d** for better comparison. **f**, Field-symmetric nonlinear second-harmonic transverse voltage ${V}_{{{{{yxx}}}},{{{\rm{sym}}}}}^{2\omega }$ versus the a.c. current amplitude ${I}_{{{{{x}}}}}^{\omega }$ at fixed values of *B* (Extended Data Fig. 6). The solid lines are quadratic fits.

The absence of linear conductivity makes this configuration the ideal regime to investigate the presence of nonlinear transverse responses, which are symmetry-allowed when the driving current is collinear with the magnetic field (Supplementary Note II). We have, therefore, performed systematic measurements of the second-harmonic (2*ω*) transverse responses (Fig. 3a,b) by sourcing the a.c. current along the $[\bar{1}10]$ direction. We have subsequently disentangled the field-antisymmetric ${R}_{{{{{y}}}},{{{\rm{as}}}}}^{2\omega }=\left[{R}_{{{{{y}}}}}^{2\omega }(B)\,-\,{R}_{{{{{y}}}}}^{2\omega }(-B)\right]/2$ and field-symmetric ${R}_{{{{{y,\mathrm{sym}}}}}}^{2\omega }=\left[{R}_{{{{{y}}}}}^{2\omega }(B)\,+\,{R}_{{{{{y}}}}}^{2\omega }(-B)\right]/2$ contributions, since they originate from distinct physical effects. In particular, the antisymmetric part contains a semiclassical contribution that only depends on the conventional group velocity of the carriers at the Fermi level (Supplementary Note II). Conversely, the symmetric part originates from the anomalous velocity term of the carriers. It is a purely quantum contribution and can be expressed in terms of a BCD. We observe the following features in Fig. 3a,b. The semiclassical antisymmetric contribution has a sudden onset above a characteristic magnetic field (Fig. 3a) that is sensitive to gating (Fig. 3c). The gate dependence displays a monotonic growth consistent with its physical origin. On the contrary, the symmetric contribution displays the typical non-monotonous gate and field-amplitude dependence (Fig. 3b,d) of BC-mediated effects. The gate dependence of the nonlinear symmetric contribution obtained by sourcing the current along the $[\bar{1}\bar{1}2]$ direction is instead strongly suppressed and featureless (Fig. 3e). This is consistent with a $[\bar{1}10]$-oriented BCD, which gives a vanishing response in this configuration. We note that the symmetric nonlinear transverse resistance has a characteristic quadratic current–voltage (*I*^*ω*^−*V*^2*ω*^), which—combined with the response at double the driving frequency—establishes its second-order nature (Fig. 3f).

**Fig. 4:**
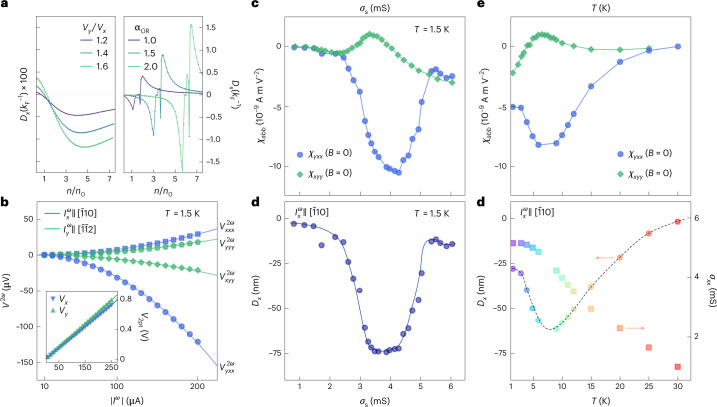
BCD under time-reversal symmetric conditions. **a**, Calculated spin-sourced and orbital-sourced BCD as a function of the sheet carrier density. The spin-sourced dipole (left) has been evaluated for different strengths of rotational symmetry-breaking distortion (∝*v*_*y*_/*v*_*x*_) (Methods), whereas the orbital-sourced dipole (right) has been computed by varying the strength of orbital Rashba coupling *α*_OR_. In both cases, the dipole has a strongly non-monotonic behaviour, goes to zero for large densities and is directed along the $[\bar{1}10]$ direction. The orbital-sourced dipole is two orders of magnitude larger in the entire density range. **b**, Measured *I*^*ω*^−*V*^2*ω*^ characteristics at zero magnetic field. Longitudinal ${V}_{{{{{xxx}}}}({{{{yyy}}}})}^{2\omega }$ and transverse ${V}_{{{{{yxx}}}}({{{{xyy}}}})}^{2\omega }$ voltage drops versus the a.c. excitation bias ∣*I*^*ω*^∣ for ${I}_{{{{{x}}}}({{{{y}}}})}^{\omega }$ along $[\bar{1}10]$ ($[\bar{1}\bar{1}2]$) at *σ*_*xx*_ ≈ *σ*_*yy*_ ≈ 4.5–4.6 mS. The solid lines are quadratic fits. The inset shows the linear two-terminal *I*–*V* characteristics highlighting the ohmic behaviour of the electrical contacts to the 2DES. The solid lines are linear fits. **c**, Sheet conductance dependence of the measured nonlinear transverse-conductivity tensor elements *χ*_*yxx*_ and *χ*_*xyy*_ for *I*^*ω*^ sourced along the two orthogonal in-plane principal crystallographic directions. **d**, BCD magnitude *D*_*x*_ under time-reversal symmetric conditions (*B* = 0). The BCD estimated using equation (1) is found to strongly peak at intermediate-doping levels, where it reaches the maximum value of nearly –75 nm. **e**, Temperature dependence of the nonlinear transverse conductivities *χ*_*yxx*_ and *χ*_*xyy*_. The two quantities go to zero as the temperature increases and strontium titanate recovers a higher (non-polar tetragonal) crystal symmetry. Concomitantly, the BCD is forced to vanish by symmetry. **f**, Temperature dependence of BCD *D*_*x*_ (left axis) and the corresponding change in sheet conductance *σ*_*xx*_(*T*) of the 2DES (right axis). The solid and dashed lines in **d** and **f** are guides to the eye.

The fact that only the symmetric contribution persists even in the zero-field limit (Fig. 3a,b) indicates the presence of a finite BCD in the absence of externally applied magnetic fields and thus of a nonlinear Hall effect in time-reversal symmetric conditions. To support the existence of a finite BCD with time-reversal symmetry, we have individually evaluated the dipole originating from the spin-sourced BC and the dipole related to the orbital-sourced BC (Methods). Figure 4a shows that in the entire parameter space of our low-energy theory model, the spin-sourced BCD is two orders of magnitude smaller than the orbital-sourced BCD. The latter exceeds the inverse characteristic Fermi wavenumber $k_\mathrm{F}^{-1}$ ≈ 0.5 nm. Besides the intrinsic contribution to the BCD, the nonlinear Hall response with time-reversal symmetry also contains disorder-induced contributions^10,42^ due to nonlinear skew and side-jump scattering. We experimentally access such contributions by measuring the longitudinal signal ${V}_{{{{{yyy}}}}}^{2\omega }$ that is symmetry-allowed but does not possess any intrinsic BCD contribution. As displayed in Fig. 4b, the strong difference in amplitude between the longitudinal signal and transverse ${V}_{{{{{yxx}}}}}^{2\omega }$ signal over a large driving-current range proves the absence of three-fold rotation symmetry as well as a nonlinear Hall effect completely dominated by the intrinsic BCD. The anisotropy between longitudinal and transverse nonlinear signals also allows us to exclude a leading role played by thermoelectric effects due to Joule heating (Fig. 4b, inset). We further observe that both longitudinal ${V}_{{{{{xxx}}}}}^{2\omega }$ and transverse ${V}_{{{{{xyy}}}}}^{2\omega }$ responses have an amplitude comparable with the longitudinal signal ${V}_{{{{{yyy}}}}}^{2\omega }$, thus suggesting their disorder-induced nature. We point out that the finite amplitudes of ${V}_{{{{{xxx}}}}}^{2\omega }$ and ${V}_{{{{{xyy}}}}}^{2\omega }$ imply ${{{{\mathcal{M}}}}}_{\bar{1}10}$ symmetry breaking (Supplementary Note II). This can be related to the mirror-breaking arrangements of the oxygen atoms caused by the antiferrodistortive octahedron rotations.^43^ It might also be due to the presence of structural domain patterns appearing at the cubic-to-tetragonal structural transition.

We have systematically verified the occurrence of a sizable nonlinear transverse response over the full range of sheet conductances and concomitantly observed a large difference between the two nonlinear transverse-conductivity tensor component *χ*_*yxx*_ and *χ*_*xyy*_ (Fig. 4c). This further proves the main intrinsic BCD contribution to the nonlinear Hall response. By further evaluating the momentum relaxation time *τ* (Supplementary Note II), we can estimate the size of the BCD (Methods):

1 $${D}_{{{{{x}}}}}=\frac{2{\hslash }^{2}}{{e}^{3}\tau }\,{\chi }_{{{{{yxx}}}}}\,.$$

The resulting BCD (Fig. 4d) is two orders of magnitude larger than the dipole observed in systems with massive Dirac fermions, such as bilayer WTe_2_ (refs. ^11,12^) and—over a finite density range—a factor of two larger than the dipole observed in corrugated bilayer graphene^13^. We attribute the large magnitude of this effect to the fact that the orbital-sourced BC is naturally equipped with a large dipolar density due to the presence of singular pinch points and hotspots with dipolar arrangements. We also monitored the temperature dependence of transverse-conductivity tensor components *χ*_*yxx*_ and *χ*_*xyy*_ (Fig. 4e) and the corresponding behaviour of BCD *D*_*x*_ (Fig. 4f). All these quantities rapidly drop approaching 30 K, that is, the temperature above which the strong polar quantum fluctuations of SrTiO_3_ vanish. This further establishes the orbital Rashba coupling as the physical mechanism behind the orbital-sourced BC.

The pure orbital-based mechanism of BCD featured here paves the way to the atomic-scale design of quantum sources of nonlinear electrodynamics persisting up to room temperature. Oxide-based 2DES could be, for instance, combined with a room-temperature polar ferroelectric layer, triggering symmetry lowering and thus inducing orbital Rashba coupling by interfacial design. This and other alternative platforms combining a low-symmetry crystal with orbital degrees of freedom and polar modes, including room-temperature polar metals^44^ and conducting ferroelectric domain walls, are candidate oxide architectures to perform operations such as rectification^45^ and frequency mixing. Moreover, multiple sources of BC can be implemented for combined optoelectronic and spintronic functionalities in a single-material system: photogalvanic currents due to the orbital-sourced BC can be employed to create spin Hall voltages exploiting the spin-sourced BC. Our study also establishes a general approach to generate topological charge distributions in strongly correlated materials, opening a vast space for exploration at the intersection between topology and correlations.

## Methods

### Sample growth

**Extended Data Fig. 7:**
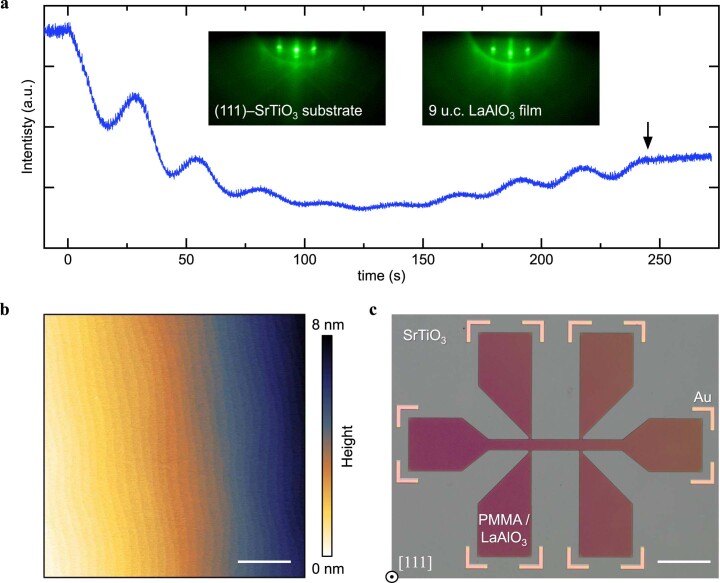
Growth, structural characterization and device fabrication of (111)-oriented LaAlO_3_/SrTiO_3_ 2DES. **a**, *In situ* real-time RHEED monitoring of the layer-by-layer PLD growth of a 9 u.c. LaAlO_3_ film on a (111)-oriented SrTiO_3_ substrate. Insets: RHEED patterns acquired before (left) and after the film growth (right), highlighting the high crystalline quality of the epitaxial LaAlO_3_ film. The vertical arrow marks the end of the growth (at 245 s). **b**, Atomic force microscopy image of a 9 u.c. thick LaAlO_3_ film on SrTiO_3_(111). The film’s topography reproduces the characteristic atomically sharp steps-and-terraces structure of the substrate’s vicinal surface. Horizontal scale bar: 1 *μ*m. **c**, Optical micrograph of a patterned PPMA resist hard mask on a 9 u.c. blanket film of LaAlO_3_/SrTiO_3_ (before the Ar ion milling step) which ultimately defines the Hall bar device area where the 2DES subsists. Pre-patterned gold markers are used to signify the position of the Hall bars’ contact pads, as the optical contrast between the etched area (bare substrate) and the crystalline LaAlO_3_/SrTiO_3_ devices is extremely low. Scale bar: 200 *μ*m.

The nine-unit-cell-thick LaAlO_3_ crystalline layer is grown on the TiO-rich surface of a (111)-oriented SrTiO_3_ substrate, from the ablation of a high-purity (>99.9%) LaAlO_3_ sintered target by pulsed laser deposition using a KrF excimer laser (wavelength, 248 nm). We perform the real-time monitoring of growth by following intensity oscillations, in a layer-by-layer growth mode, of the first diffraction spot using reflection high-energy electron diffraction (Extended Data Fig. 7a). This allows us to stop the growth at precisely the critical thickness of nine unit cells of LaAlO_3_ (ref. ^46^) necessary for the (111)-oriented LaAlO_3_/SrTiO_3_ 2DES to form. The SrTiO_3_(111) substrate was first heated to 700 °C in an oxygen partial pressure of 6 × 10^−5^ mbar. The LaAlO_3_ layer was grown in those conditions at a laser fluence of 1.2 J cm^−2^ and laser repetition rate of 1 Hz. Following the growth of the LaAlO_3_ layer, the temperature is ramped down to 500 °C before performing one-hour-long in situ annealing in a static background pressure of 300 mbar of pure oxygen, to recover the oxygen stoichiometry of the reduced heterostructure. Finally, the sample is cooled down at –20 °C min^−1^, and kept in the same oxygen environment at zero heating power for at least 45 min.

### Device fabrication

The (111)-oriented LaAlO_3_/SrTiO_3_ blanket films were lithographically patterned into two Hall bars (width *W* = 40 μm; length *L* = 180 μm), oriented along the two orthogonal crystal-axis directions of $[\bar{1}10]$ and $[\bar{1}\bar{1}2]$. The Hall bars are defined by electron-beam lithography into a poly(methyl methacrylate) resist, which is used as a hard mask for argon-ion milling (Extended Data Fig. 7c). The dry-etching duration is calibrated and timed to be precisely stopped when the LaAlO_3_ layer is fully removed to avoid the creation of an oxygen-deficient conducting SrTiO_3−δ_ surface. This leaves an insulating SrTiO_3_ matrix surrounding the protected LaAlO_3_/SrTiO_3_ areas, which host a geometrically confined 2DES.

### Electrical transport measurements

The Hall bars are connected to a chip carrier by an ultrasonic wedge-bonding technique in which the aluminium wires form ohmic contacts with the 2DES through the LaAlO_3_ overlayer. The sample is anchored to the chip carrier by homogeneously coating the backside of the SrTiO_3_ substrate with silver paint. A d.c. voltage *V*_g_ is sourced between the silver back-electrode and the desired Hall-bar device to enable electrostatic-field-effect gating of the 2DES, leveraging the large dielectric permittivity of strontium titanate at low *T* (~2 × 10^4^ below 10 K)^47,48^. Non-hysteretic dependence of *σ*_*xx*_ (*σ*_*yy*_) on *V*_g_ is achieved following an initial gate-forming procedure^49^.

Standard four-terminal electrical (magneto-)transport measurements were performed at 1.5 K in a liquid helium-4 flow cryostat, equipped with a superconducting magnet (maximum magnetic field, *B* = ±12 T). An a.c. excitation current *I*^*ω*^ ∝ |*I*^*ω*^|sin(*ω*t), of frequency *ω*/(2π) = 17.77 Hz, is sourced along the desired crystallographic direction. The sheet resistance, ${R}_{{{{\rm{s}}}}}={\sigma }_{{{{{xx}}}}}^{-1}$, of a Hall-bar device is related to the first-harmonic longitudinal voltage drop *V*_*xx*_ according to *R*_s_ = (*V*_*xx*_/*I*_*x*_)(*W*/*L*). When the a.c. current is sourced along $\hat{{{{\bf{x}}}}}\parallel [\bar{1}10]$ ($\hat{{{{\bf{y}}}}}\parallel [\bar{1}\bar{1}2]$), we make use of a standard lock-in detection technique to concomitantly measure the first-harmonic longitudinal response *V*_*xx*_ (*V*_*yy*_), and either the in-phase first-harmonic ${V}_{{{{{xy}}}}}^{\omega }$ (${V}_{{{{{yx}}}}}^{\omega }$) or out-of-phase second-harmonic ${V}_{{{{{yxx}}}}}^{2\omega }$ (${V}_{{{{{xyy}}}}}^{2\omega }$) transverse voltages (Fig. 1a). We define the first- and second-harmonic transverse resistances as ${R}_{{{{{xy}}}}}^{\omega }={V}_{{{{{xy}}}}}^{\omega }/| {I}_{{{{{x}}}}}^{\omega }|$ and ${R}_{{{{{y}}}}}^{2\omega }={V}_{{{{{yxx}}}}}^{2\omega }/| {I}_{{{{{x}}}}}^{\omega }{| }^{2}$, respectively. First- and second-harmonic measurements are performed at 10 and 50 μA, respectively.

We systematically decompose both first- and second-harmonic magneto-responses into their field-symmetric ${R}_{{{{\rm{sym}}}}}^{(2)\omega }$ and field-antisymmetric ${R}_{{{{\rm{as}}}}}^{(2)\omega }$ contributions according to

2a $${R}_{{{{\rm{sym}}}}}^{(2)\omega }=\left[{R}^{(2)\omega }(B)+{R}^{(2)\omega }(-B)\right]/2\,,$$

2b $${R}_{{{{\rm{as}}}}}^{(2)\omega }=\left[{R}^{(2)\omega }(B)-{R}^{(2)\omega }(-B)\right]/2\,.$$

In particular, the first-harmonic transverse resistance is purely field antisymmetric, and hence, we chose the simplified notation of ${R}_{{{{{xy}}}}}\equiv {R}_{{{{{xy}}}},{{{\rm{as}}}}}^{\omega }$.

### Estimation of the Rashba spin–orbit energy from magnetoconductance measurements in the weak antilocalization regime

In a 2DES, in the presence of a spin relaxation mechanism induced by an additional spin–orbit interaction, the conductance is subject to weak localization corrections at lower temperatures. Extended Data Fig. 4a shows the gate-modulated magnetoconductance curves of the 2DES, which exhibit a characteristic low-field weak antilocalization behaviour. The magnetoconductance curves, normalized to the quantum of conductance *G*_Q_ = *e*^2^/(π*ħ*), are fitted using a Hikami–Larkin–Nagaoka model that expresses the change in conductivity Δ*σ*(*B*_⟂_) = *σ*(*B*_⟂_) – *σ*(0) of the 2DES under an external out-of-plane magnetic field *B*_⊥_, in the diffusive regime (with negligible Zeeman splitting), as follows^50,51^:

3 $$\begin{array}{ll}\frac{{{\Delta }}\sigma ({B}_{\perp })}{{G}_{{{{\rm{Q}}}}}}&=-\frac{1}{2}{{\varPsi }}\left(\frac{1}{2}+\frac{{B}_{{{{\rm{i}}}}}}{{B}_{\perp }}\right)+\frac{1}{2}\ln \left(\frac{{B}_{{{{\rm{i}}}}}}{B}\right)\\ &+{{\varPsi }}\left(\frac{1}{2}+\frac{{B}_{{{{\rm{i}}}}}+{B}_{{{{\rm{so}}}}}}{{B}_{\perp }}\right)-\ln \left(\frac{{B}_{{{{\rm{i}}}}}+{B}_{{{{\rm{so}}}}}}{{B}_{\perp }}\right)\\ &+\frac{1}{2}{{\varPsi }}\left(\frac{1}{2}+\frac{{B}_{{{{\rm{i}}}}}+2{B}_{{{{\rm{so}}}}}}{{B}_{\perp }}\right)-\frac{1}{2}\ln \left(\frac{{B}_{{{{\rm{i}}}}}+2{B}_{{{{\rm{so}}}}}}{{B}_{\perp }}\right)\\ &-{A}_{{{{{\rm{K}}}}}}\frac{\sigma (0)}{{G}_{{{{\rm{Q}}}}}}{B}_{\perp }^{2}\,\end{array},$$

where *Ψ* is the digamma function; *ħ* = *h*/(2π) is the reduced Planck constant; ${B}_{{{{\rm{i}}}},{{{\rm{so}}}}}=\hslash /\left(4eD{\tau }_{{{{\rm{i}}}},{{{\rm{so}}}}}\right)$ are the effective fields related to the inelastic and spin–orbit relaxation times (*τ*_i_ and *τ*_so_, respectively); and *D* = π*ħ*^2^*σ*(0)/(*e*^2^m*) is the diffusion constant. The last term in equation (3), proportional to ${B}_{\perp }^{2}$, contains *A*_K_, the so-called Kohler coefficient, which accounts for orbital magnetoconductance.

Hence, from the fit to the weak antilocalization magnetoconductance curves, the effective Rashba spin–orbit coupling *α*_R_ can be calculated as

4 $${\alpha }_{{{{\rm{R}}}}}={\hslash }^{2}/\left[2{m}^{* }\sqrt{\left(D{\tau }_{{{{\rm{so}}}}}\right)}\right]\,,$$

based on a D’yakonov–Perel’ spin relaxation mechanism^51^. A summary of the dependence of the extracted parameters on the 2DES’ sheet conductance is plotted in Extended Data Fig. 5b. The spin–orbit energy *Δ*_so_ can then be estimated according to

5 $${{{\varDelta }}}_{{{{\rm{so}}}}}=2{\alpha }_{{{{\rm{R}}}}}{k}_{{{{\rm{F}}}}}\,,$$

where, in two dimensions, the Fermi wavevector is given by ${k}_{{{{\rm{F}}}}}=\sqrt{2\uppi {n}_{2{{{\rm{D}}}}}}$, assuming a circular Fermi surface. The sheet carrier density *n*_2D_ is experimentally obtained for each doping value from the (ordinary) Hall effect (Supplementary Note III), measured concomitantly with the magnetoconductance traces.

### Spin-sourced and orbital-sourced BCD calculations

We first estimate the BCD due to spin sources in time-reversal symmetry condition as a function of carrier density considering the low-energy Hamiltonian for a single Kramers’-related pair of bands (Supplementary Note I):

6 $${{{\mathcal{H}}}}=\frac{{{{{\bf{k}}}}}^{2}}{2m({{{\bf{k}}}})}-{\alpha }_{{{{\rm{R}}}}}\,{{{\bf{\sigma }}}}\cdot {{{\bf{k}}}}\times \hat{{{{\bf{z}}}}}+\frac{\lambda }{2}({k}_{+}^{3}+{k}_{-}^{3}){\sigma }_{{{{{z}}}}},$$

where the momentum-dependent mass can be negative close to the Γ point (Supplementary Note I). Although this model Hamiltonian is equipped with a finite BC, its dipole is forced to vanish by the three-fold rotation symmetry (Supplementary Note I). We capture the rotation symmetry breaking of the low-temperature structure at the leading order by assuming inequivalent coefficients for the spin–orbit coupling terms linear in momentum. In other words, we make the substitution *α*_R_(*σ*_*x*_*k*_*y*_ – *σ*_*y*_*k*_*x*_)→*v*_*y*_*k*_*y*_*σ*_*x*_ – *v*_*x*_*k*_*x*_*σ*_*y*_. Since the dipole is a pseudo-vector, the residual mirror symmetry ${{{{\mathcal{M}}}}}_{x}$ forces it to be directed along the $\hat{{{{\bf{x}}}}}$ direction. In the relaxation-time approximation, it is given by

7 $${D}_{x}={\int}_{{{{\bf{k}}}}}{\partial }_{{k}_{x}}{{{\varOmega }}}_{{{{{z}}}}}({{{\bf{k}}}}),$$

where *Ω*_*z*_ is the BC of our two-band model that we write in a dimensionless form by measuring energies in units of ${k}_{{{{\rm{F}}}}}^{2}/2m({k}_{{{{\rm{F}}}}})$, lengths in units of 1/*k*_F_ and densities in units of ${n}_{0}={k}_{{{{\rm{F}}}}}^{2}/2\uppi$. Here *k*_F_ is a reference Fermi wavevector. For simplicity, we have considered a positive momentum-independent effective mass. For the BCD shown in Fig. 4a, the remaining parameters have been chosen as *v*_*x*_ = 0.4, *v*_*y*_ = (1.2, 1.4, 1.6) × *v*_*x*_ and *λ* = 0.1. Moreover, we account for orbital degeneracy by tripling the dipole of a single Kramers’ pair. This gives an upper bound for the spin-sourced BCD.

We have also evaluated the BCD due to orbital sources considering the low-energy Hamiltonian for spin–orbit-free *t*_2g_ electrons derived from symmetry principles (Supplementary Note I) and reading

8 $$\begin{array}{ll}{{{\mathcal{H}}}}({{{\bf{k}}}})=&\frac{{\hslash }^{2}{{{{\bf{k}}}}}^{2}}{2m}{{{\varLambda }}}_{0}+{{\varDelta }}\left({{{\varLambda }}}_{3}+\frac{1}{\sqrt{3}}{{{\varLambda }}}_{8}\right)+{{{\varDelta }}}_{{{{{m}}}}}\left(\frac{1}{2}{{{\varLambda }}}_{3}-\frac{\sqrt{3}}{2}{{{\varLambda }}}_{8}\right)\\ &-{\alpha }_{{{{\rm{OR}}}}}\left[{k}_{{{{{x}}}}}{{{\varLambda }}}_{5}+{k}_{{{{{y}}}}}{{{\varLambda }}}_{2}\right]-{\alpha }_{{{{{\rm{m}}}}}}{k}_{{{{{x}}}}}{{{\varLambda }}}_{7}\end{array},$$

where we introduced the Gell–Mann matrices as

$$\begin{array}{l}{{{\varLambda }}}_{2}=\left(\begin{array}{rcl}0&-i&0\\ i&0&0\\ 0&0&0\end{array}\right){{{\varLambda }}}_{3}=\left(\begin{array}{rcl}1&0&0\\ 0&-1&0\\ 0&0&0\end{array}\right)\\ {{{\varLambda }}}_{5}=\left(\begin{array}{rcl}0&0&-i\\ 0&0&0\\ i&0&0\end{array}\right){{{\varLambda }}}_{7}=\left(\begin{array}{rcl}0&0&0\\ 0&0&-i\\ 0&i&0\end{array}\right)\\ {{{\varLambda }}}_{8}=\left(\begin{array}{lll}\frac{1}{\sqrt{3}}&0&0\\ 0&\frac{1}{\sqrt{3}}&0\\ 0&0&\frac{-2}{\sqrt{3}}\end{array}\right)\end{array},$$

and *Λ*_0_ is the identity matrix. In the Hamiltonian above, *Δ* is the splitting between the *a*_1g_ singlet and ${e}_{\mathrm{g}}^{{\prime} }$ doublet resulting from the *t*_2g_ orbitals in a trigonal crystal field. Here *Δ*_*m*_ is the additional splitting between the doublet caused by rotational symmetry breaking. Finally, *α*_OR_ and *α*_m_ are the strengths of the orbital Rashba coupling. Note that in the presence of three-fold rotation symmetry, *α*_m_ ≡ 0, in which case the BC is forced to vanish. For simplicity, we have evaluated the BC for the ${{{{\mathcal{C}}}}}_{\mathrm{s}}$ point group-symmetric case assuming *α*_m_ ≡ *α*_OR_. In our continuum SU(3) model, the BC can be computed using the method outlined elsewhere^52^. We have subsequently computed the corresponding dipole measuring, as before, energies in units of ${k}_{{{{\rm{F}}}}}^{2}/2m$, lengths in units of 1/*k*_F_ and densities in units of ${n}_{0}={k}_{{{{\rm{F}}}}}^{2}/2\uppi$. The dimensionless orbital Rashba coupling has been varied between *α*_OR_ = 1 and *α*_OR_ = 2, whereas we have fixed *Δ* = –0.1 and *Δ*_*m*_ = 0.005. The value of the crystal field splitting *Δ* is consistent with the amplitude determined by X-ray absorption spectroscopy^53^ of the order 8 meV, and therefore, it is almost one order of magnitude smaller than our energy unit of ~40 meV for a reference ${k}_{\mathrm{F}}^{-1}$ ≃ 0.5 nm and effective mass *m* ≃ 3*m*_e_ (Supplementary Note III). The calculated dipole (Fig. 4a) has been finally multiplied by two to account for spin degeneracy. As shown in Supplementary Note I, we remark that the model Hamiltonian for the spin sources of BC (equation (6)) and the model Hamiltonian for the orbital sources (equation (8)) derive from a single six-band model where orbital and spin degrees of freedom are treated on an equal footing.

### Estimation of BCD magnitude from nonlinear Hall measurements

The nonlinear current density is mathematically given by ${j}_{\alpha }^{2\omega }={\chi }_{\alpha \beta \gamma }\,{E}_{\beta }\,{E}_{\gamma }$, where *χ*_*αβγ*_ is the nonlinear transverse-conductivity tensor. When an a.c. current density ${I}_{{{{{x}}}}}^{\omega }/W={\sigma }_{{{{{xx}}}}}{E}_{{{{{x}}}}}^{\omega }$ is sourced along $\hat{{{{\bf{x}}}}}$, the second-harmonic transverse current density developing along $\hat{{{{\bf{y}}}}}$ is related to the BCD **D** according to^9^

9 $${{{{\bf{j}}}}}_{{{{{y}}}}}^{2\omega }\,=\,\frac{{e}^{3}\tau }{2{\hslash }^{2}(1+\mathrm{i}\omega \tau )}\left(\hat{{{{\bf{z}}}}}\times {{{{\bf{E}}}}}_{{{{{x}}}}}^{\omega }\right)\left({{{\bf{D}}}}\cdot {{{{\bf{E}}}}}_{{{{{x}}}}}^{\omega }\right)\,,$$

where *τ* is the momentum relaxation time and *e* is the elementary charge. Due to the mirror symmetry ${{{{\mathcal{M}}}}}_{{{{{x}}}}}\equiv {{{{\mathcal{M}}}}}_{[\bar{1}10]}$, the dipole is found to point along $\hat{{{{\bf{x}}}}}$; in the quasi-d.c. limit, that is, (*ωτ*) ≪ 1, the BCD expression reduces to

10 $${D}_{{{{{x}}}}}=\frac{2{\hslash }^{2}}{{e}^{3}\tau }\frac{{j}_{{{{{y}}}}}^{2\omega }}{{\left({E}_{{{{{x}}}}}^{\omega }\right)}^{2}}=\frac{2{\hslash }^{2}}{{e}^{3}\tau }\frac{{V}_{{{{{yxx}}}}}^{2\omega }\,{\sigma }_{xx}^{3}\,W}{| {I}_{{{{{x}}}}}^{\omega }{| }^{2}},$$

which is the explicit expression for equation (1), in terms of experimentally measurable quantities only, and where

11a $${\chi }_{{{{{yxx}}}}}=\frac{{j}_{{{{{y}}}}}^{2\omega }}{{\left({E}_{{{{{x}}}}}^{\omega }\right)}^{2}},$$

11b $${\chi }_{{{{{xyy}}}}}=\frac{{j}_{{{{{x}}}}}^{2\omega }}{{\left({E}_{{{{{y}}}}}^{\omega }\right)}^{2}},$$

are the measured nonlinear transverse-conductivity tensor elements shown in Fig. 4c,e.

## Online content

Any methods, additional references, Nature Portfolio reporting summaries, source data, extended data, supplementary information, acknowledgements, peer review information; details of author contributions and competing interests; and statements of data and code availability are available at 10.1038/s41563-023-01498-0.

## Supplementary information

Supplementary Information Supplementary Notes I–III and Figs. 1–14.

## Figures and Tables

**Extended Data Fig. 1:**
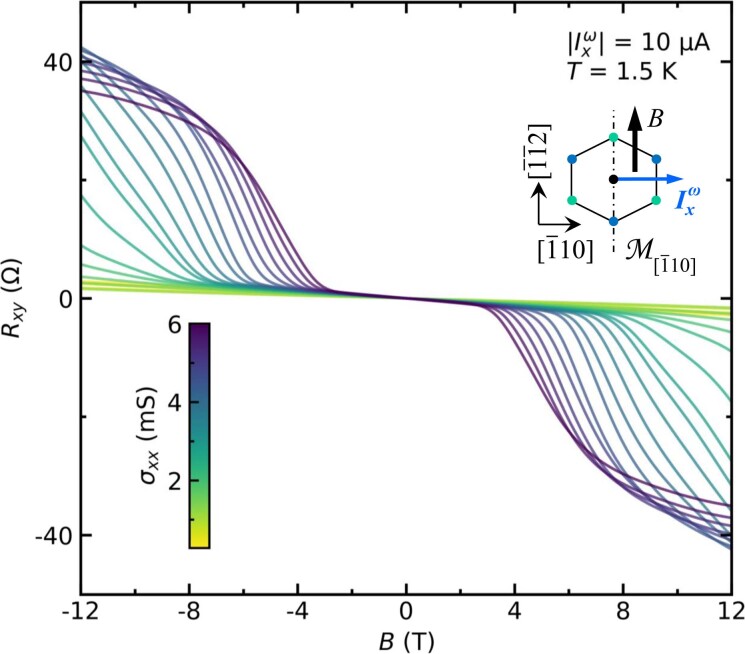
Hall effect in a planar magnetic field. Experimentally measured field-antisymmetric transverse magnetoresistance ${R}_{{{{\rm{yx}}}}}={V}_{{{{\rm{yx}}}}}^{\omega }/{I}_{{{{\rm{y}}}}}^{\omega }$ at *T* = 1.5 K, with ${I}_{{{{\rm{x}}}}}^{\omega }$ along $[\bar{1}10]\perp B$ (see inset schematic), for varying sheet conductance values *σ*_xx_ (indicated by the inset colored scale bar), and tuned via electrostatic field effect in a back-gate geometry.

**Extended Data Fig. 2:**
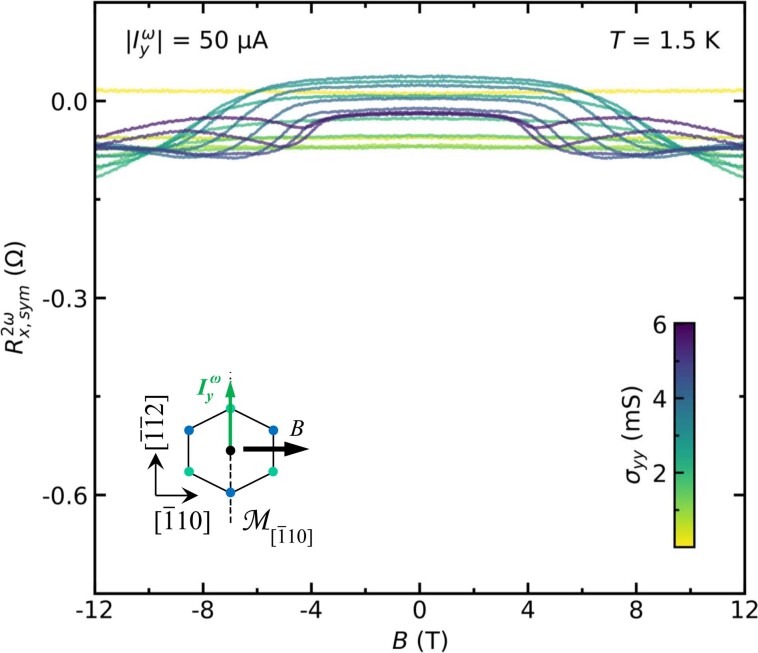
Nonlinear Hall response in a planar magnetic field. Experimentally measured field-symmetric nonlinear transverse magnetoresponse ${R}_{{{{\rm{x}}}}}^{2\omega }$ at *T* = 1.5 K with ${I}_{{{{\rm{y}}}}}^{\omega }$ along $[\bar{1}\bar{1}2]\perp B$ (see inset schematic), for varying sheet conductance values *σ*_yy_ (indicated by the inset colored scale bar). The full scale ordinate axis is chosen to be the same as for panel **b** of Fig. 3, for better comparison, and highlights the comparatively small nonlinear Hall response when the current is sourced along the ${{{{\mathcal{M}}}}}_{\bar{1}10}$ mirror line corresponding to a symmetry demanded zero BCD.

**Extended Data Fig. 6:**
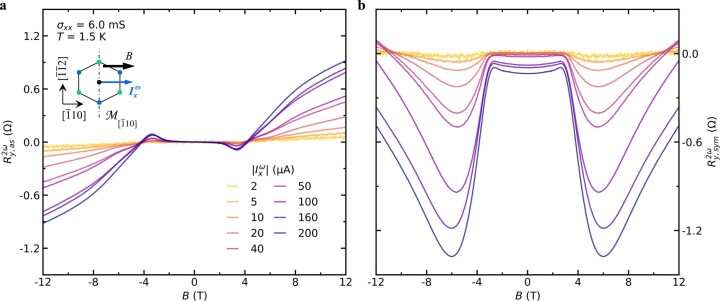
Current bias dependent nonlinear transverse signal in a planar magnetic field. **a**,**b**, Field-antisymmetric, ${R}_{{{{\rm{y}}}},{{{\rm{as}}}}}^{2\omega }$, and field-symmetric ${R}_{{{{\rm{y,sym}}}}}^{2\omega }$ second harmonic transverse resistance responses, for various magnitudes of the excitation a.c. current along $[\bar{1}10]$ (∥*B*, see inset schematic).

## Data Availability

The data that support the findings of this study are available via Zenodo at 10.5281/zenodo.7575479.

## Code availability

The code that support the findings of the study is available from the corresponding authors on reasonable request.
