# Supplementary material for: Designing spin and orbital sources of Berry curvature at oxide interfaces
Source: Nat Mater. 2023 Mar 16;22(5):576–82. doi: 10.1038/s41563-023-01498-0 (PMC10156604; doi:10.1038/s41563-023-01498-0)
Supplement: Supplementary file 1 — Supplementary Notes I–III and Figs. 1–14. [file 41563_2023_1498_MOESM1_ESM.pdf]

nature materials

Article

https://doi.org/10.1038/s41563-023-01498-0

Designing spin and orbital sources of Berry curvature at oxide interfaces

In the format provided by the authors and unedited

Contents

I. Supplementary Note I: Berry curvature at trigonal oxide interfaces 1

II. Supplementary Note II: Magnetotransport theory 5
Anomalous planar Hall effect 5
Nonlinear transverse response with planar magnetic fields 8
Symmetry constraints on the linear and nonlinear resistivity tensor 8
Planar magnetoresistance computation 9

III. Supplementary Note III: Additional magnetotransport measurements 10
Ordinary Hall effect & estimation of the momentum relaxation time − gate dependence 10
Ordinary Hall effect & estimation of the momentum relaxation time − temperature dependence 12
Additional doping-dependent Hall effect measurements and magnetoconductance in the weak antilocalization regime 13
Estimation of the critical magnetic field and corresponding Zeeman energy. Comparison with the Rashba spin-orbit energy 13
Out-of-plane misalignment of the planar magnetic field 14
Angular dependence of planar magnetoresponses 17

References 18

I. Supplementary Note I: Berry curvature at trigonal oxide interfaces

To derive the electronic properties of the two-dimensional system realized at the $[111]-\mathrm{LaAlO_3/SrTiO_3}$ interface in the high-temperature trigonal phase, we first discuss the orbital and symmetry character of the electronic levels at the center of the Brillouin zone. Let us first neglect spin-orbit coupling. Due to the trigonal coordination, the three t_{2g} Ti orbitals are split into an a_{1g} orbital representing a real one-dimensional irreducible representation of the $\mathscr{C}_{3v}$ point group, and an e'_g doublet forming a real two-dimensional irreducible representation of the group. The e_g Ti orbitals are not affected by the trigonal crystal field as they also form a doublet. Let us now include the effect of spin-orbit coupling breaking the $SU(2)$ spin rotation symmetry. The two spin-orbit coupled states originating from the a_{1g} orbital have a symmetry-protected degeneracy since they form the Γ_4 two-dimensional irreducible representation of the $\mathscr{C}_{3v}$ double-point group. Spin-orbit coupling instead splits the quartet of states originating from the e'_g doublets. Specifically, two spin-orbit coupled orbitals are singly degenerate and form the Γ_5 and the Γ_6 one-dimensional irreducible representations of the double point group. The remaining two states are instead degenerate and form a Γ_4 representation. Since the Γ_5 and Γ_6 representations are complex, time-reversal symmetry implies that these states must stick together thus forming a Kramers' doublet. The irreducible two-dimensional Γ_4 representation is instead quaternionic and therefore is already equipped with time-reversal invariance. As a result, we have that all levels at the BZ center correspond to an effective spin-$\frac{1}{2}$ Kramers' doublet.

The minimal model Hamiltonian close to each of these Kramers' doublets can be derived in a $\mathbf{k}\cdot\mathbf{p}$ expansion accounting for all symmetry-allowed terms. To do so, we note that in the surface Kramers' doublet basis the time-reversal symmetry can be represented as $\mathscr{T} = i\sigma_y\mathscr{K}$ with $\mathscr{K}$ the complex conjugation. The mirror symmetry is instead represented by $\mathscr{M} = i\sigma_x$. Note that from here onwards $\hat{x}$ will indicate the $[\bar{1}10]$ direction. In the basis $|\psi^{\uparrow\downarrow}\rangle$ the threefold rotation operator takes the form $\mathscr{C}_3 = e^{-i\sigma_z\pi/3}$. Under the operation of $\mathscr{C}_3$ and $\mathscr{M}$, momentum and spin transform as follows,

$$\begin{array}{llll} \mathscr{C}_3: & k_\pm \to e^{\pm i2\pi/3}k_\pm, & \sigma_\pm \to e^{\pm i2\pi/3}\sigma_\pm, & \sigma_z \to \sigma_z \\ \mathscr{M}: & k_+ \to -k_- & \sigma_x \to \sigma_x, & \sigma_{y,z} \to -\sigma_{y,z} \end{array}$$

where $k_\pm = k_x \pm ik_y$ and $\sigma_\pm = \sigma_x \pm i\sigma_y$. The Hamiltonian must also be invariant under time reversal which adds the constraint $\mathscr{H}(\mathbf{k}) = \mathscr{T}\mathscr{H}(-\mathbf{k})\mathscr{T}^{-1} = \sigma_y\mathscr{H}^*(-\mathbf{k})\sigma_y$.

At linear order in the momentum $\mathbf{k}$, and including an effective regularizing quadratic term, the minimal two-band Hamiltonian for a Kramers' related pair of bands reads:

$$\mathscr{H}_\mathrm{R}(\mathbf{k}) = \frac{\mathbf{k}^2}{2m}\sigma_0 - \alpha_\mathrm{R}\,\boldsymbol{\sigma}\cdot\mathbf{k}\times\hat{\mathbf{z}}, \tag{1}$$

where $\boldsymbol{\sigma}$ is a vector of Pauli matrices, σ_0 is the identity matrix,$\alpha_{\rm R}$ is the “Rashba" spin-orbit coupling strength, while m is the effective electron mass. Note that nothing prevents the effective electron mass to be negative. In this case, electron-like transport can be ensured by adding a term quartic in momentum $\mathbf{k}^4/(2m_1)\,\sigma_0$ with $m_1>0$. Overall, this amounts to consider a momentum dependent effective electron mass. The Hamiltonian in Eq. (1) does not capture crystalline anisotropy effects. In addition, the Berry curvature associated to this minimal model is zero, since there is no term proportional to σ_z. However, higher order momentum terms change this situation. The first symmetry allowed term accounting for crystalline anisotropy is third order in momentum and takes the form,

$$\mathscr{H}_w(\mathbf{k}) = \frac{\lambda}{2}(k_+^3+k_-^3)\sigma_z\,. \tag{2}$$

This warping Hamiltonian is proportional to the Pauli matrix σ_z, which is crucial to obtain a non-zero Berry curvature and leads to out-of-plane spin textures. Note that since the full Hamiltonian is invariant under the mirror symmetry $\mathscr{M}$, $\mathscr{H}_w(\mathbf{k})$ is forced to vanish along the mirror line.

To show the presence of a finite Berry curvature induced by warping, we recall that in a two band model the Berry curvature can be calculated by rewriting the full Hamiltonian as $\mathscr{H}(\mathbf{k}) = \mathbf{d}(\mathbf{k})\cdot\boldsymbol{\sigma}+\mathbf{k}^2\sigma_0/[2m(\mathbf{k})]$, where $\mathbf{d}$ is a momentum dependent vector, which for our specific model has components $\mathbf{d}=\left\{-k_y,k_x,\lambda\left(k_+^3+k_-^3\right)/2\right\}$. The $\mathbf{d}$ vector is independent of terms $\propto\sigma_0$, and thus of the momentum dependent effective electron mass. The expression for the Berry curvature is then given by $\Omega_z^{\pm}(\mathbf{k}) = \pm\hat{\mathbf{d}}\cdot(\partial_{k_x}\hat{\mathbf{d}}\times\partial_{k_y}\hat{\mathbf{d}})/2$ with $\hat{\mathbf{d}}=\mathbf{d}/|\mathbf{d}|$. For our minimal model Hamiltonian in the presence of trigonal symmetry, we have

$$\Omega_{\pm}^{z}(k,\theta) = \pm\frac{2\sqrt{2}\lambda\,\alpha_{\rm R}^2k^3\cos(3\theta)}{\left[2\alpha_{\rm R}^2k^2+\lambda^2k^6\cos(6\theta)+\lambda^2k^6\right]^{3/2}}\,, \tag{3}$$

where θ is the polar angle in momentum space.

The Berry curvature is well defined in each point except the origin where the bands are degenerate. Note that the constraints set by time reversal symmetry and the three-fold rotational symmetry are satisfied as can be verified upon a closer inspection of Eq. (3). Moreover $\Omega_{\pm}^{z}(k,\theta)$ vanishes along the mirror lines, in accordance with Eq. (2).

The orbital sources of Berry curvature can be instead derived by explicitly considering interorbital mixing terms. To this end, it is convenient to neglect spin-orbit coupling. The effective Hamiltonian for the a_{1g} and e_g' doublet in the trigonal crystal field can be derived using symmetry principles. Specifically, any generic 3×3 Hamiltonian can be expanded in terms of the nine Gell-Mann matrices Λ_i as

$$\mathscr{H}(\mathbf{k}) = \sum_{i=0}^{8} b_i(\mathbf{k})\Lambda_i\,. \tag{4}$$

The invariance of the Hamiltonian requires that the components of the Hamiltonian vector $\mathbf{b}(\mathbf{k})$ should have the same behavior as the corresponding Gell-Mann matrices Λ_i. This means that they should belong to the same representation of the crystal point group. The representation of the Gell-Mann matrices Λ_i and those of the polynomials of $\mathbf{k}$ can be found using that the generators of the point group for spinless electrons can be written as

$$\mathscr{M}_x = \begin{pmatrix} 1 & 0 & 0\\ 0 & 1 & 0\\ 0 & 0 & -1\end{pmatrix};\quad \mathscr{C}_3 = \begin{pmatrix} 1 & 0 & 0\\ 0 & \cos\frac{2\pi}{3} & \sin\frac{2\pi}{3}\\ 0 & -\sin\frac{2\pi}{3} & \cos\frac{2\pi}{3}\end{pmatrix}.$$

With this, the effective Hamiltonian up to linear order in momentum reads

$$\mathscr{H}(\mathbf{k}) = \frac{\mathbf{k}^2}{2m}\Lambda_0+\Delta\left(\Lambda_3+\frac{1}{\sqrt{3}}\Lambda_8\right)-\alpha_{\rm OR}\left[k_x\Lambda_5+k_y\Lambda_2\right], \tag{5}$$

where Λ_0 is the identity matrix and the other Gell-Mann matrices are reported in the main text. The last term in the equation above corresponds to the orbital Rashba coupling with strength $\alpha_{\rm OR}$. The second term gives the crystal field splitting of size Δ between the a_{1g} singlet and the e_g' doublet. As discussed in the main text, a direct computation of the Berry curvature using the method outlined in Ref. 1 yields a vanishing Berry curvature at all momenta. This changes by considering a crystalline symmetry lowering to $\mathscr{C}_s$. From the representation of Gell-Mann matrices and polynomials of momentum, the effective Hamiltonian now takes the form

$$\mathscr{H}_{\rm OR}(\mathbf{k}) = \frac{\mathbf{k}^2}{2m}\Lambda_0+\Delta\left(\Lambda_3+\frac{1}{\sqrt{3}}\Lambda_8\right)+\Delta_m\left(\frac{1}{2}\Lambda_3-\frac{\sqrt{3}}{2}\Lambda_8\right)-\alpha_{\rm OR}\left[k_x\Lambda_5+k_y\Lambda_2\right]-\alpha_m k_x\Lambda_7\,.$$

In the equation above, the crystal field splitting Δ_m yields an energy separation between the e'_g states at the center of the BZ. This Hamiltonian is characterized by Berry curvature distributions with dipolar hot spots and singular pinch points. Furthermore, it is naturally equipped with a large non-vanishing Berry curvature dipole. The dispersion of the bands is strongly dependent on the ratio between the characteristic orbital Rashba energy $E_{\text{OR}} = 2m\alpha_{\text{OR}}^2$ and the crystal field splittings. In particular, the effective mass of the lowest band is negative close to the Γ point of the BZ whenever the orbital Rashba energy is comparable to the crystal-field splitting Δ. In this regime, an intraband Lifshitz transition occurs when increasing the chemical potential. On the contrary, in the opposite regime of large crystal field splittings, all bands possess an effective electron mass that is positive at all momenta, and consequently intraband Lifshitz transitions do not take place. This is explicitly shown in Fig. S1 where we report the evolution of the bands by continuously increasing the Δ/E_{OR} ratio. Note that similar features are also found within the remaining doublet of bands. In this case the momentum dependence of the effective mass is controlled by the ratio between the crystal field splitting Δ_m and the effective orbital Rashba energy $E_{\text{OR}} = 2m\alpha_m^2$, and can acquire a non-trivial dependence along the $k_y = 0$ line.

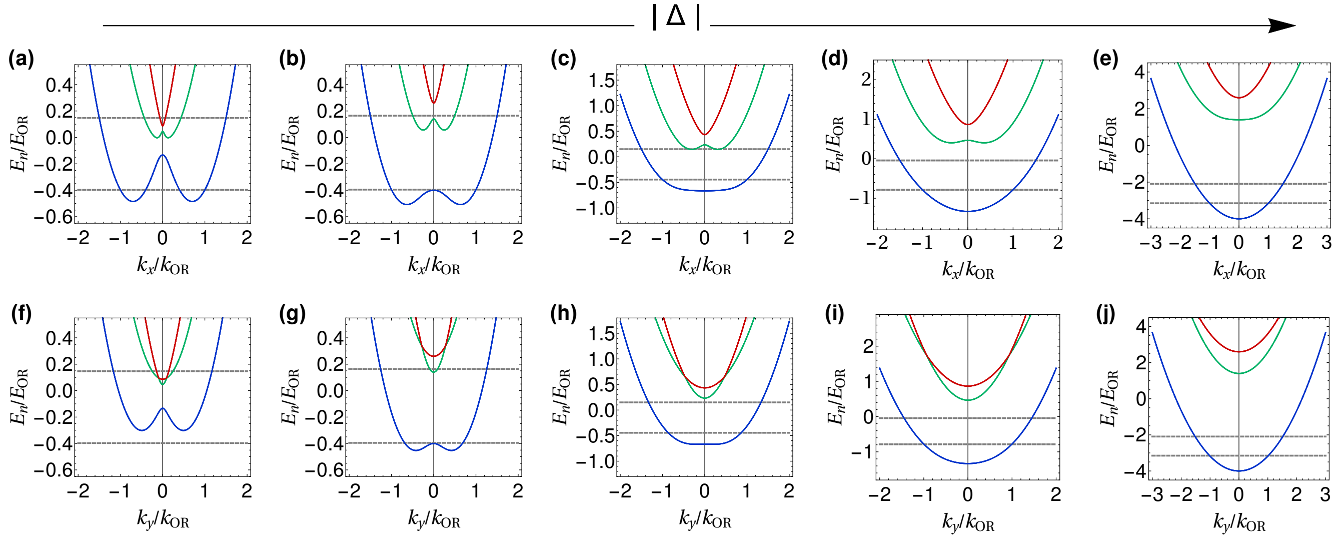

Figure S 1. **Dependence of the orbital-sourced model on crystal field splittings.** Evolution of the energy bands on the $k_y = 0$ line (top panels) and the $k_x = 0$ line (bottom panels) for the spin-orbit-free orbital-sourced model by increasing the ratio between the crystal field splitting Δ and the characteristic orbital Rashba energy $E_{\text{OR}} = 2m\alpha_{\text{OR}}^2$. The panels correspond to a) $\Delta/E_{\text{OR}} = -0.1$, (b) $\Delta/E_{\text{OR}} = -0.3$, (c) $\Delta/E_{\text{OR}} = -0.5$, (d) $\Delta/E_{\text{OR}} = -1.0$, and (e) $\Delta/E_{\text{OR}} = -3.0$. Same values are used for the sequence (f)-(j). The crystal field splitting $\Delta_m = 0.2\,|\Delta|$ and we have chosen the orbital Rashba parameter $\alpha_m = \alpha_{\text{OR}}$. All energies are measured in units of E_{OR} whereas momenta are measured in units of the orbital Rashba momentum $k_{\text{OR}} = 2m\alpha_{\text{OR}}$. The gray lines are upper and lower bounds for the Fermi energy.

In Fig. S1 we also indicate the relevant range of energies in the band structure, which we evaluate using the following argument. To obtain a Berry curvature dipole of order k_F, the orbital Rashba parameter has to be comparable to $k_F/(2m)$. This is explicitly shown in Fig. 3a of our main manuscript – the Berry curvature dipole has a sweet spot and can exceed $1/k_F$ when $2m\alpha_{OR} \simeq k_F$. Hence, the orbital Rashba momentum $k_{OR} \simeq k_F$. Considering the finite range of the Berry curvature dipole sweet spot and the additional tuning of the carrier density by gating (see Supplementary Note III), we estimate that the Fermi wavevector $k_{OR} < k_F < 1.5k_{OR}$. This consequently gives an upper and lower bounds for the Fermi energy shown as gray lines in Fig. S1. We note that the possibility of occupying more than one band is in line with the observations presented in Ref. 2. Finally we wish to point out that since the orbital Rashba energy corresponds to the characteristic kinetic energy $k_F^2/(2m)$, which lies in the tens of meV range, all values of the crystal field splitting Δ considered in Fig. S1 could be realized in practice – x-ray absorption spectroscopy [3] indicates a crystal field splitting of the order 8 meV. In other words, the material could be on the verge of a change in the sign of the effective mass close to zero momentum.

We next show that both the Hamiltonian characterizing the orbital degrees of freedom and the Hamiltonian in Eq. (1) for the spin degree of freedom can be derived from a single model that treats orbital and spin degrees of freedom on an equal footing. In order to make a link between the spin and orbital degrees of freedom one needs to include the atomic spin-orbit coupling.

Then, the starting point for the coupled spin and orbital degrees of freedom is provided by the following model Hamiltonian:

$$\mathscr{H}_{tot}(\mathbf{k}) \;=\; \mathscr{H}_{\text{OR}}(\mathbf{k}) \otimes \sigma_0 + \lambda_{\text{so}}(L_x \otimes \sigma_x + L_y \otimes \sigma_y + L_z \otimes \sigma_z), \tag{6}$$

where we have included the atomic spin-orbit coupling with the strength λ_{so} and σ_i indicate the spin Pauli matrices with $i = x, y, z$. We recall that the orbital angular momentum projected on the effective subspace with the three selected orbital configurations

has the following representation in terms of the Gell-Mann matrices: $L_x = \Lambda_2$, $L_y = \Lambda_5$, and $L_z = \Lambda_7$.

For simplicity, we next consider a $\mathscr{C}_{3v}$ symmetric (i.e. $\Delta_m = \alpha_m = 0$) crystalline environment. At the Γ point of the BZ, and explicitly including atomic spin-orbit coupling, the Hamiltonian reduces to:

$$\mathscr{H}_{tot}^{\Gamma} = \mathscr{H}_{\mathrm{OR}}^{\Gamma} \otimes \sigma_0 + \lambda_{\mathrm{so}}(L_x \otimes \sigma_x + L_y \otimes \sigma_y + L_z \otimes \sigma_z), \tag{7}$$

with

$$\mathscr{H}_{\mathrm{OR}}^{\Gamma} = \Delta\left(\Lambda_3 + \frac{1}{\sqrt{3}}\Lambda_8\right). \tag{8}$$

$\mathscr{H}_{tot}^{\Gamma}$ can be diagonalized and yields the following double degenerate (Kramer pairs) eigenvalues $E_A = \tilde{E_A} + \frac{4}{3}\Delta$,$E_B = \tilde{E_B} + \frac{4}{3}\Delta$, $E_C = \tilde{E_C} + \frac{4}{3}\Delta$ with:

$$\tilde{E}_C = \frac{1}{2}\left(-2\Delta - \lambda_{\mathrm{so}} + \sqrt{4\Delta^2 + 4\Delta\lambda_{\mathrm{so}} + 9\lambda_{\mathrm{so}}^2}\right) \tag{9}$$

$$\tilde{E}_B = -2\Delta + \lambda_{\mathrm{so}} \tag{10}$$

$$\tilde{E}_A = \frac{1}{2}\left(-2\Delta - \lambda_{\mathrm{so}} - \sqrt{4\Delta^2 + 4\Delta\lambda_{\mathrm{so}} + 9\lambda_{\mathrm{so}}^2}\right). \tag{11}$$

According to the character of the trigonal splitting and the Hund's rule (spin-orbit coupling favors the highest total angular momentum at low filling) λ_{so} and Δ have negative values. Thus, the following energy relation holds $E_A < E_B < E_C$. E_A is the lowest energy in the multiplet.

The eigenvectors can be also written in a compact vectorial form as:

$$|v_{C,1}\rangle = \frac{1}{\sqrt{2+c^2}}[-c,0,0,-i,0,1] \tag{12}$$

$$|v_{C,2}\rangle = \frac{1}{\sqrt{2+c^2}}[0,c,i,0,1,0] \tag{13}$$

$$|v_{B,1}\rangle = \frac{1}{\sqrt{2}}[0,0,0,i,0,1] \tag{14}$$

$$|v_{B,2}\rangle = \frac{1}{\sqrt{2}}[0,0,-i,0,1,0] \tag{15}$$

$$|v_{A,1}\rangle = \frac{1}{\sqrt{2+a^2}}[-a,0,0,-i,0,1] \tag{16}$$

$$|v_{A,2}\rangle = \frac{1}{\sqrt{2+a^2}}[0,a,i,0,1,0], \tag{17}$$

with

$$a = -\frac{\tilde{E}_C}{\lambda_{\mathrm{so}}} \tag{18}$$

$$c = -\frac{\tilde{E}_A}{\lambda_{\mathrm{so}}}. \tag{19}$$

In the new spin-orbit coupled basis the Hamiltonian $\mathscr{H}_{tot}$ has the following matrix form:

$$\mathscr{H}_{tot} = \begin{pmatrix} H_{CC} & H_{CB} & H_{CA} \\ H_{CB}^{\dagger} & H_{BB} & H_{BA} \\ H_{CA}^{\dagger} & H_{BA}^{\dagger} & H_{AA} \end{pmatrix} \tag{20}$$

with H_{MN} being 2x2 matrices.

Let us now focus on the matrix elements of the block Hamiltonian H_{AA} associated with the lowest energy doublet assuming that the energy splitting among the levels is larger than the strength occurring in the inter-doublet terms (i.e. H_{CB},H_{BA}, H_{CA}). We emphasize that since the energy splitting between the spin-orbit Kramers' doublets is controlled (at fixed values of the crystal fields) by the spin-orbit coupling strength, the intradoublet hybridization channels H_{BA}, H_{CA}, H_{CB} can be safely neglected in the

large spin-orbit coupling regime $\lambda_{\rm so} \gg E_{\rm OR}$. In this case, one can directly determine the effective spin Hamiltonian by evaluating the following expectation values:

$$H_{AA}(i,j) = \langle v_{A,i} | \mathscr{H}_{tot} | v_{A,j} \rangle. \tag{21}$$

Taking into account the expressions of the eigenvectors $v_{A,i}$ and of the k-dependent terms of the total Hamiltonian, the matrix of the block H_{AA} can be written as:

$$H_{AA} = E_A + \frac{\mathbf{k}^2}{2m} + \begin{pmatrix} \frac{\lambda}{2}(k_+^3 + k_-^3) & -\alpha_{\rm R}(ik_x - ky) \\ -\alpha_{\rm R}(-ik_x - ky) & -\frac{\lambda}{2}(k_+^3 + k_-^3) \end{pmatrix} \tag{22}$$

with the effective intra-doublet Rashba interaction given by

$$\alpha_{\rm R} = \frac{2\alpha_{\rm OR}\lambda_{\rm so}}{\sqrt{4\Delta^2 + 4\Delta\lambda_{\rm so} + 9\lambda_{\rm so}^2}}. \tag{23}$$

Note that in the Hamiltonian above the trigonal warping in the spin sector is found by equipping the orbital model Hamiltonian with a symmetry-allowed cubic term $\propto \gamma(k_+^3 + k_-^3)\Lambda_7$. Then the warping coupling λ has the following expression

$$\lambda = \gamma \frac{16\lambda_{\rm so}^2}{8\lambda_{\rm so}^2 + \tilde{E}_A^2}. \tag{24}$$

Therefore, the effective low-energy spin model can be directly linked to the orbital model with its sources of Berry curvature dipole. Two remarks are in order here. First, using a Löwdin procedure one can verify that there exist higher-order corrections, *e.g.* momentum dependent renormalization of the Rashba coupling $\alpha_{\rm R} \to \alpha_{\rm R}(1 + \delta\mathbf{k}^2)$. Second, the bare effective electron mass appearing in Eq. (22) is corrected by intraorbital mixing terms due to the finiteness of the spin-orbit coupling strength. This generally results in a momentum dependent mass that can be negative close to zero momentum in agreement with the features of the spin-orbit free orbital bands discussed above. This is explicitly proven in Fig. S2 where we show the evolution of the spin-orbit coupled bands by increasing $\lambda_{\rm so}$. Spin-orbit coupling generally weakens the momentum dependence of the effective mass eventually leading to a positive effective mass at all momenta. Importantly, and as mentioned above, the symmetry-based spin model can be applied without restrictions even in the presence of a strongly momentum dependent mass. Therefore, the spin sources of Berry curvature are correctly captured even for intermediate spin-orbit coupling strenghts.

In this case, the spin-sourced model can be applied up to momenta for which the corresponding energy of the spin bands is comparable to the splitting ΔE_Γ between two different Kramers doublets at the center of the BZ. This gives an upper bound for the Fermi wavevector $k_{\rm F} = \sqrt{k_{\rm R}^2/4 + m\Delta E_\Gamma} - k_{\rm R}/2$ with $k_{\rm R} = 2m\alpha_{\rm R}$.

Our experimental observations indicate the occurrence of a strong anomalous planar Hall effect at the same carrier densities at which a sizable non-linear Hall effect at zero magnetic field is measured. The characteristic features of the anomalous planar Hall effect can be explained using the spin-sourced model – the orbital-sourced Berry curvature is stiff in response to externally applied in-plane magnetic fields. Instead, the non-linear Hall effect at zero magnetic field cannot be quantitatively captured within this picture, and the orbital sources of Berry curvature are needed. Putting these observations together, we can therefore conclude that both orbital Rashba and spin-orbit coupling are relevant ingredients to describe the physical properties of the material structure. Our experimental tools independently probe the Berry curvature associated with orbital and spin degrees of freedom.

II. Supplementary Note II: Magnetotransport theory

Anomalous planar Hall effect

In this section we discuss the Hall effect induced by the spin-sourced Berry curvature in the presence of a planar magnetic field using a semiclassical Boltzmann framework.

We first note that the presence of a planar magnetic field generates a Zeeman coupling term in the low-energy Hamiltonian $\mathscr{H}_B = B(\sigma_x \cos\phi + \sigma_y \sin\phi)$, that breaks $\mathscr{C}_3$ when present, $\mathscr{T}$ and the remaining mirror $\mathscr{M}_x$ except when the direction of the magnetic field with respect to the $\hat{x}$ axis $\phi = 2\pi m/6$ with $m \in \mathbb{N}$. In this case one mirror symmetry is preserved. The energy dispersion of the total Hamiltonian is then given by:

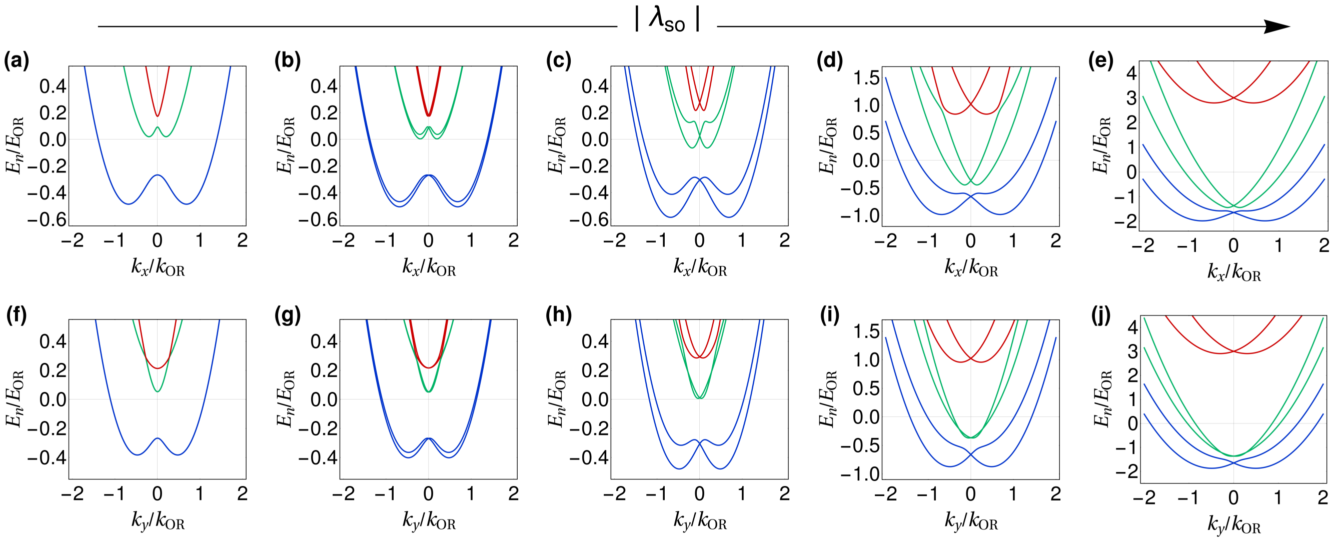

Figure S 2. **Dependence of the energy bands on spin-orbit coupling.** Evolution of the energy bands on the $k_y = 0$ line (top panels) and the $k_x = 0$ line (bottom panels) for the full model Eq. (6) by increasing the ratio between the spin-orbit coupling strength $\lambda_{\rm so}$ and the characteristic orbital Rashba energy $E_{\rm OR} = 2m\alpha_{\rm OR}^2$. The panels correspond to (a) $\lambda_{\rm so} = 0$, (b) $\lambda_{\rm so}/E_{\rm OR} = -0.02$, (c) $\lambda_{\rm so}/E_{\rm OR} = -0.1$, (d) $\lambda_{\rm so}/E_{\rm OR} = -0.5$, and (e) $\lambda_{\rm so}/E_{\rm OR} = -1.5$. Same values are used for the sequence (f)-(j). The crystal field splittings have been chosen as $\Delta = -0.2E_{\rm OR}$ and $\Delta_m = 0.2|\Delta|$. Finally the orbital Rashba parameter $\alpha_m = \alpha_{\rm OR}$. All energies are measured in units of $E_{\rm OR}$ whereas momenta are measured in units of the orbital Rashba momentum $k_{\rm OR} = 2m\alpha_{\rm OR}$.

$$\begin{aligned}\varepsilon_\pm(\mathbf{k}) \;=\; & \frac{\mathbf{k}^2}{2m} + \left[(B\sin(\phi)+k_x\alpha_{\rm R})^2 + (k_y\alpha_{\rm R} - B\cos(\phi))^2 \right. \\ & \left. +\lambda^2\left(k_x^3 - 3k_xk_y^2\right)^2\right]^{1/2}. \qquad (25)\end{aligned}$$

The planar magnetic field breaks the Kramer's degeneracy at the center of the BZ and completely splits the two bands. However, when a mirror is preserved there is a mirror-symmetry protected Dirac point along the mirror line at $(k,\theta) = (|B|/\alpha_{\rm R}, \phi + sgn(B)\pi/2)$. The condition $k_F = |B|/\alpha_{\rm R}$ defines the critical magnetic field strength introduced in the main text. Moreover, a Lifshitz transition (a change in topology in the Fermi surface, in this case going from one closed Fermi line to two) appears since the Zeeman coupling shifts the minima of the two bands. The energy for which the upper band is completely depleted is given by $\varepsilon_L = B^2/(2m\alpha_{\rm R}^2)$ if $B < m\alpha_{\rm R}^2$ otherwise $\varepsilon_L = B - m\alpha_{\rm R}^2/2$ if $B > m\alpha_{\rm R}^2$.

To derive the transport characteristic associated to this low-energy model we assume to apply an electric field $\mathbf{E} = E\hat{\mathbf{x}}$ along the $\hat{x}$ axis and rotate the magnetic field in the $x-y$ plane. Thus we write the magnetic field as $\mathbf{B} = B(\cos\phi\hat{\mathbf{x}} + \sin\phi\hat{\mathbf{y}})$. Generally speaking, the semiclassical equations of motions in the presence of a non-zero Berry curvature read

$$\dot{\mathbf{r}} = D(\mathbf{B},\Omega_k)[\mathbf{v_k} + e(\mathbf{E}\times\Omega_k) + e(\mathbf{v_k}\cdot\Omega_k)\mathbf{B}] \qquad (26)$$

$$\dot{\mathbf{k}} = D(\mathbf{B},\Omega_k)[e\mathbf{E} + e(\mathbf{v_k}\times\mathbf{B}) + e^2(\mathbf{E}\cdot\mathbf{B})\Omega_k], \qquad (27)$$

where $D(\mathbf{B},\Omega_k) = [1+(e)(\mathbf{B}\cdot\Omega_k)]^{-1}$, Ω_k is the Berry curvature and $\mathbf{v_k}$ is the group velocity. Solving the Boltzmann equation for the electron distribution function $f(k)$ within the relaxation time approximation allows to compute the charge current $\mathbf{J} = e\int(d^dk/(2\pi)^d)\dot{\mathbf{r}}f(k)D^{-1}$ that accounts for the modified phase space factor D. In linear response theory the charge current obeys the relation $J_a = \sigma_{ab}E_b$, where σ_{ab} are the components of the conductivity tensor and E_b the external electric field. Following Ref. 4, the planar Hall conductivity, after discarding higher order contributions, is given by

$$\begin{aligned}\sigma_{yx} \;=\; & e^2\int\frac{d^dk}{(2\pi)^d}D\tau\left(-\frac{\partial f_{eq}}{\partial\varepsilon}\right)\{[v_y + eB\sin\theta(\mathbf{v_k}\cdot\Omega_k)] \\ & [v_x + eB\cos\theta(\mathbf{v_k}\cdot\Omega_k)]\} + \frac{e^2}{h}\int\frac{d^2k}{(2\pi^2)}\Omega_k^z f_{eq}, \qquad (28)\end{aligned}$$

where f_{eq} is the equilibrium Fermi-Dirac distribution. Eq. (28) contains all transverse linear responses in the presence of coplanar electric and magnetic fields in both two- and three-dimensional systems. In three-dimensional topological Dirac and type-I Weyl semimetals, the Berry-curvature induced planar Hall effect stems from the term $\propto B^2 \sin\theta \cos\theta (\mathbf{v_k} \cdot \Omega_k)^2$. The last term instead represents the usual anomalous Hall contribution for magnetic materials. In two-dimensional systems, $\mathbf{v_k} \perp \Omega_k$. However, in trigonal crystals a non-vanishing contribution to the last term of Eq. (28) appears once the Zeeman interaction is explicitly taken into account, and exists provided all mirror symmetries are broken, even though the material is non-magnetic. This also implies that, contrary to three-dimensional topological semimetals, the Berry-curvature related planar Hall effect of two-dimensional systems does not explicitly depend on the relative angle between the electric and magnetic field but only on the angle between the planar magnetic field and the principal crystallographic directions. This, in turns, allows to directly observe a purely antisymmetric planar Hall effect when the electric and magnetic fields are perfectly aligned since in this configuration the classical contribution of Eq. (28), containing the $v_x v_y$ term vanishes.

We have computed the behavior of the anomalous planar Hall conductivity assuming a magnetic field direction $\phi = \pi/2$. In this configuration all mirror symmetries are broken. Moreover the integral of the Berry curvature is maximum. The contribution to the linear transverse response σ_{xy} is given by the integral of the Berry curvature over the Fermi surfaces of the two energy bands. Since the two bands contribute with opposite curvatures it is sufficient to integrate over the exclusion region of the two bands. Hence the anomalous contribution to the transverse conductivity, at zero temperature is given by

$$\sigma_{xy} \propto \int_{\mathscr{S}_+} \Omega_z^+(\mathbf{k}) + \int_{\mathscr{S}_-} \Omega_z^-(\mathbf{k}) = \int_{\mathscr{S}_+ \cap \mathscr{S}_-} \Omega_z^-(\mathbf{k}), \tag{29}$$

where $\mathscr{S}_\pm$ are the Fermi surfaces of the two bands and the last integral contains Ω^- since it is the curvature of the outermost band. The magnetic field dependence at constant density can be calculated numerically by varying the Fermi energy as the magnetic field is changed. Indeed, by keeping the area of the surface $\mathscr{S}_+ \cap \mathscr{S}_-$ fixed the number of electronic carriers stays constant. To obtain the resistivity ρ_{xy} it is necessary to compute the two longitudinal conductivities σ_{xx} and σ_{yy}. In the relaxation time approximation these are given by,

$$\sigma_{\alpha\alpha} = e^2 \tau \sum_{\gamma=\pm} \int_{\mathbf{k}} (\partial_\alpha \varepsilon_\gamma)^2 (-\frac{\partial f_0}{\partial \varepsilon_\gamma}), \tag{30}$$

where $\alpha = (x, y)$, ε_γ is the energy dispersion of the band γ and f_0 is the equilibrium Fermi-Dirac distribution. The resistivity ρ_{xy} is then obtained by inverting the conductivity tensor,

$$\rho_{xy} = \frac{\sigma_{xy}}{\sigma_{xx}\sigma_{yy} + \sigma_{xy}^2}. \tag{31}$$

Here we have used that the transverse conductivity is purely antisymmetric, *i.e.* $\sigma_{xy}(B) = -\sigma_{yx}(B)$, since the semiclassical contributions are vanishing.

Figure S3 shows the corresponding behavior of the anomalous planar Hall resistivity as a function of the magnetic field strength for different values of the carrier density. The plot has been obtained by writing the low-energy Hamiltonian in dimensionless form measuring energies units of $k_F^2/2m$, lengths in units of $1/k_F$ and density in units of $n_0 = k_F^2/2\pi$. The remaining dimensionless parameters have been fixed to $\alpha_R = 0.4$, and $\lambda = 0.1$ Furthermore, we have used the approximate expression for the transverse resistivity $\rho_{xy} \simeq \sigma_{xy}/(\sigma_{xx}\sigma_{yy})$, which is accurate since the transverse conductivity is much smaller than the linear in τ longitudinal resistivity. We obtain that the anomalous planar Hall resistivity increases non-monotonically and gets enhanced by decreasing the carrier density. Note that when considering the observed decrease of the carrier density as the sheet conductivity increases (see Supplementary Note VI), this trend is in agreement with the behavior of the antisymmetric Hall resistance reported in the main text.

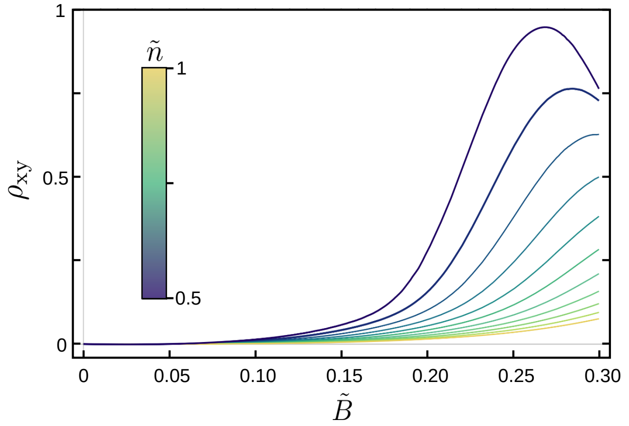

Figure S 3. **Calculated planar Hall resistivity versus band filling.** Calculated magnetic field dependence of the anomalous planar Hall resistivity (measured in arbitrary units) ρ_{xy}, for different carrier densities. The magnetic field $\tilde{B}$ is normalized in units of $k_F^2/2m$ and the densities $\tilde{n}$ are in units of $k_F^2/2\pi$. The anomalous planar Hall contribution is maximum for values of the magnetic field that shift the anti-crossing point close to the Fermi energy, and goes back to zero for stronger magnetic fields.

Nonlinear transverse response with planar magnetic fields

Nonlinear transverse currents have two intrinsic contributions: the first is a semiclassical term that depends on the integral of the electronic velocities, whereas the other has a purely quantum nature stemming from the Berry curvature dipole:

$$\sigma^{\text{sc}}_{\alpha\alpha\beta} \propto \int_k \partial^2_\alpha f_0 \partial_\beta \varepsilon_k \tag{32}$$

$$\sigma^{\text{dip}}_{\alpha\alpha\beta} \propto \varepsilon_{\alpha\beta} \int_k (\partial_\beta \Omega) f_0 \,, \tag{33}$$

where $\varepsilon_{\alpha\beta}$ is the Levi-Civita antisymmetric tensor, $\partial_\alpha = \partial_{k_\alpha}$, f_0 is the equilibrium Fermi Dirac distribution and ε_k is the energy dispersion. The two conductivities are the proportionality factors between the applied AC electric field $(E^\omega_\alpha)^2$ and the second harmonic response $j^{2\omega}_\beta$. Typically the full response is measured, and it is necessary to decouple the two contributions in order to extract the magnitude of the Berry curvature dipole. This can be done by considering how the two conductivities behave when switching the sign of the magnetic field. The $\sigma^{\text{sc}}_{\alpha\alpha\beta}$ is odd in B: this can be seen by sending $B \to -B$ and applying the coordinate change $\mathbf{k} \to -\mathbf{k}$. While the integration measure $\partial k_x \partial k_y$ and the f_0 remain unchanged $[\varepsilon(\mathbf{k}, B) = \varepsilon(-\mathbf{k}, -B)]$ the three derivatives bring an overall minus sign. On the other hand the contribution $\sigma^{\text{dip}}_{\alpha\alpha\beta}$ is even with respect to the sign of the magnetic field. Sending $B \to -B$ and $\mathbf{k} \to -\mathbf{k}$ produces a sign change in the Berry curvature $[\Omega(\mathbf{k}, B) = -\Omega(-\mathbf{k}, -B)]$ which is compensated by the minus sign originating from the coordinate change in the partial derivative.

Symmetry constraints on the linear and nonlinear resistivity tensor

We recall that the linear conductivity tensor is defined by the relation

$$j_\alpha = \sigma_{\alpha\beta} E_\beta \,. \tag{34}$$

We can derive the transformation rule of the conductivity tensor under a generic point group symmetry represented by an orthogonal matrix $\mathscr{O}$ by simply noticing that both the current j and the driving electric field E transform as vectors under a generic coordinate change. Therefore, the conductivity tensor transforms as $\mathscr{O}^T \sigma \mathscr{O}$. For the point group $\mathscr{C}_s$ the single mirror symmetry $\mathscr{M}_x$ implies that the transverse conductivity $\sigma_{xy} \equiv \sigma_{yx} \equiv 0$. In crystals with $\mathscr{C}_{3v}$ point group symmetry instead,

the additional threefold rotation symmetry implies that the two longitudinal conductivities along the principal crystallographic directions $\sigma_{xx} \equiv \sigma_{yy}$. Crystalline symmetries also pose constraints on the nonlinear conductivity tensor defined by

$$j_\alpha = \chi_{\alpha\beta\gamma} E_\beta E_\gamma . \qquad (35)$$

The transformation rule of the nonlinear conductivity tensor imply that in the presence of a $\mathscr{M}_x$ mirror symmetry, we have $\chi_{xxx} = \chi_{xyy} = \chi_{yxy} = \chi_{yyx} = 0$. The additional threefold rotation symmetry in the $\mathscr{C}_{3v}$ point group symmetry implies that the non-zero component of the nonlinear conductivity tensor satisfy the relation $\chi_{xxy} = \chi_{xyx} = \chi_{yxx} = -\chi_{yyy}$. A violation of this relation implies that the trigonal symmetry is broken and only a mirror symmetry is present in the system.

Planar magnetoresistance computation

We have computed the planar magnetoresistance MR $= [\rho_{xx,yy}(B)/\rho_{xx,yy}(0) - 1]$ considering a planar magnetic field directed along the $[\bar{1}10]$ direction, thus preserving the mirror symmetry. In this case, the transverse Berry-mediated conductance σ_{xy} vanishes. The magnetoresistance is strongly anisotropic and indeed a qualitative difference exists depending on whether the driving current is collinear or orthogonal to the magnetic field. Specifically in the former case (see Fig. S4) a small positive magnetoresistance starts to develop when the Fermi energy crosses the mirror symmetry-protected Dirac point (orange line). This positive magnetoresistance persists up to the Lifshitz point (red line). After the Lifshitz transition, the magnetoresistance starts decreasing and reaches a negative saturation value as also seen in experiments (see Fig. S5). The negative magnetoresistance can be intuitively understood by considering that after the Lifshitz transition scattering between the two energy bands is suppressed, hence enhancing the magnetoconductance. In the configuration in which the driving electric field and the magnetic field are orthogonal, the magnetoresistance is always negative and the weak-field positive magnetoresistance does not occur [see Fig. S2(a)]. This is in agreement with the features observed at the $LaVO_3$-$KTaO_3$ interface [5]. Note that for configurations in which the magnetic field is not mirror-preserving, a similar type of behaviour is still expected since the quantum contribution is a lower order correction to the semiclassical one.

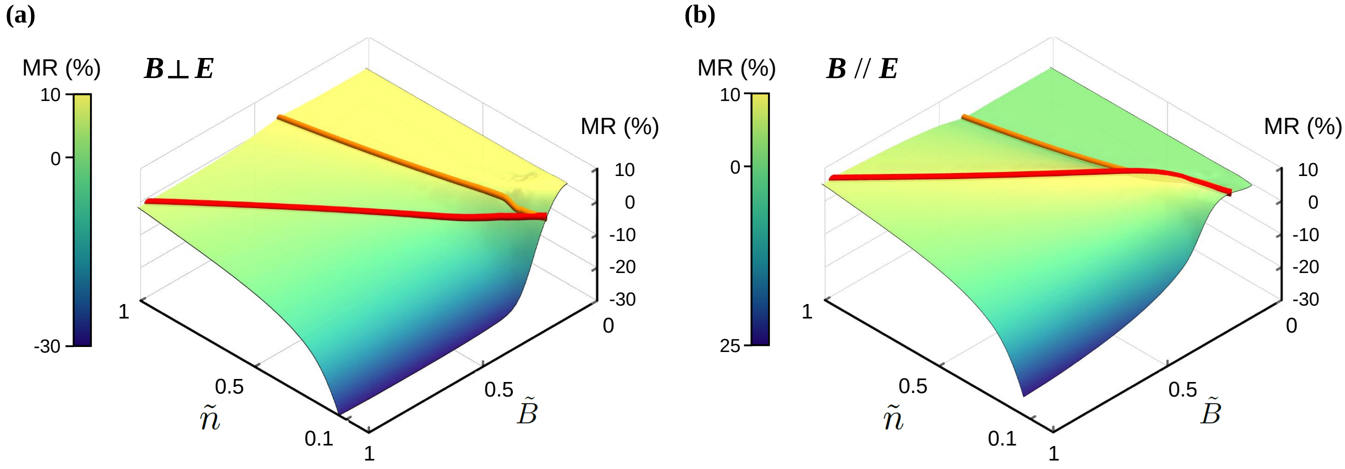

Figure S 4. **Calculated planar magnetoresistance.** Planar magnetoresistance as obtained by considering a planar magnetic field in the $[\bar{1}10]$ direction and an orthogonal **(a)**, or collinear **(b)** driving electric field. Since the magnetic field is mirror-symmetry preserving $\rho_{xx,yy} = 1/\sigma_{xx,yy}$. The magnetoresistance has been obtained using the same parameter set as in Fig. S1, and is shown also as a function of the carrier density. The magnetic field $\tilde{B}$ is normalized in units of $k_F^2/2m$ and the densities $\tilde{n}$ are in units of $k_F^2/2\pi$.

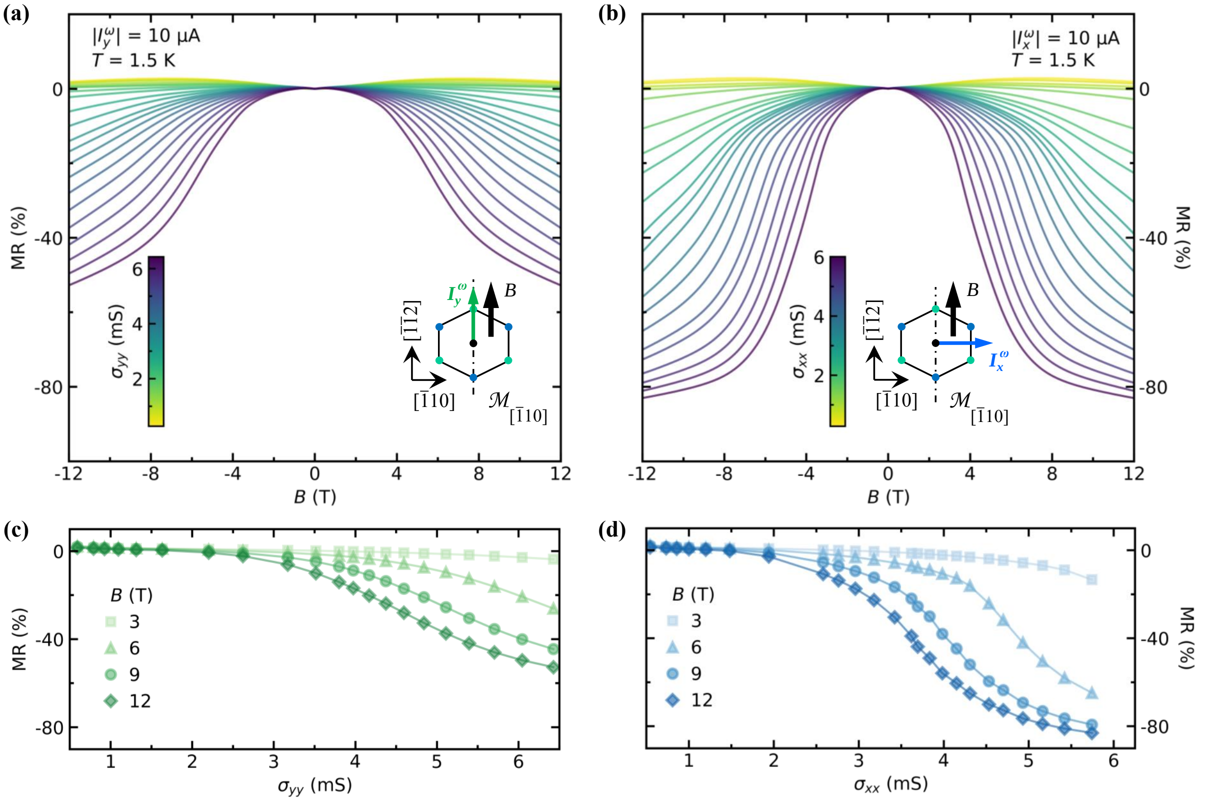

Figure S 5. **Measured gate-dependent planar magnetoresistance.** Gate modulated magnetoresistance in an in-plane magnetic field for I_y^ω along $[\bar{1}\bar{1}2]$ parallel to B **(a)**, and for I_x^ω along $[\bar{1}10]$ transverse to B **(b)**. In both cases, the MR is seen to grow negatively above a critical planar magnetic field value. Panels **(c)** and **(d)** display the corresponding sheet conductance dependences of the MR at constant magnetic field values. For both Hall bar devices, the MR shows an onset above a given value of sheet conductance followed by a monotonic increase, and even an apparent saturation for the curve corresponding to $B = 12$ T.

III. Supplementary Note III: Additional magnetotransport measurements

Ordinary Hall effect & estimation of the momentum relaxation time − gate dependence

Figure S6(a,b) display the gate-modulated ordinary Hall effect, and longitudinal MR (respectively) acquired in the same device, oriented along $[\bar{1}10]$, presented throughout the manuscript; with $B_\perp$ the out-of-plane magnetic field. At low doping levels, the two-dimensional electron system (2DES) exhibits a linear Hall effect, while the low-field MR is indicative of a weak-antilocalization regime, as reported previously [2, 6]. At sheet conductance values σ_{xx} exceeding ≈ 2 mS, $\rho_{xy}(B_\perp)$ is found to depart from a purely linear Hall effect. Non-linearities in the ordinary Hall effect response have been discussed extensively in the framework of a multi-carrier conduction, or multi-orbital conduction when considering $SrTiO_3$-based 2DES [2, 7, 8]. Here, in the case of the [111]$-LaAlO_3/SrTiO_3$ 2DES, the nonlinear Hall component has been attributed to the populating of replica sub-bands of the t_{2g}-manifold (derived from the Ti-3d bands) in the quantum well [2, 9].

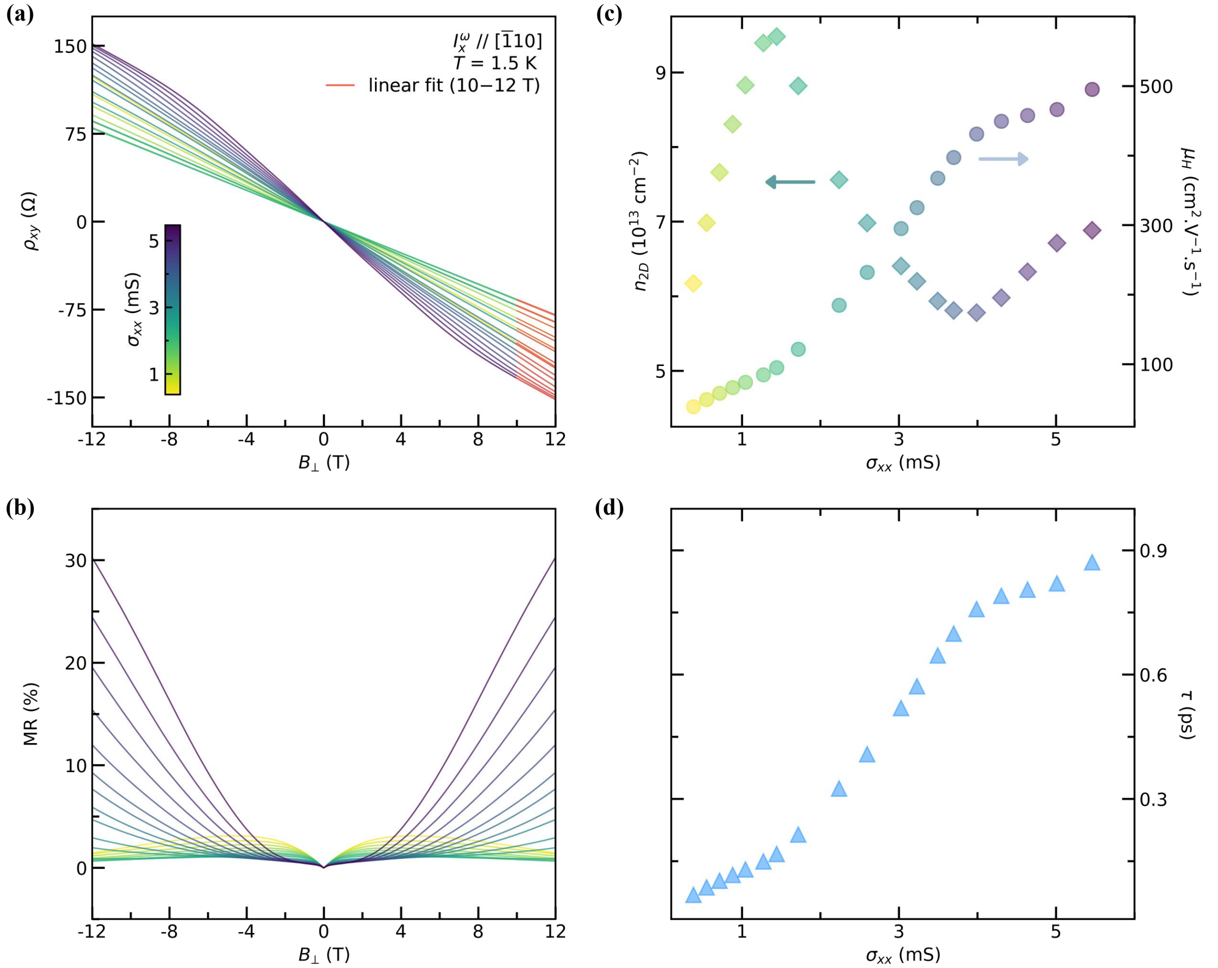

Figure S 6. **Ordinary Hall effect and magnetoresistance of the 2DES.** **(a)** Gate-modulated ordinary Hall effect response of the 2DES for the current along $[\bar{1}10]$. Solid red lines are linear fits to $\rho_{xy}(B_\perp)$ performed between 10 and 12 T. **(b)** Corresponding longitudinal magnetoresistance. $B_\perp$, the out-of-plane magnetic field. **(c)** Experimentally estimated sheet carrier density n_{2D} (left axis) and electronic mobility μ_H (right axis) as a function of doping levels. **(d)** Momentum relaxation time τ versus sheet conductance σ_{xx}, obtained in a Drude model, following Eq. (38)

A number of authors have further discussed the relevance of the two-band model for the determination of meaningful transport parameters values, *i.e.*, carrier densities and mobilities [2, 7, 8]. We simply point out, that in the limit of large magnetic fields, the total carrier density in the system, which we denote n_{2D}, can be related to the slope of ρ_{xy} vs. $B_\perp$ via:

$$n_{2D} = \frac{-1}{eR_H}. \tag{36}$$

where $R_H = \partial\rho_{xy}(B_\perp)/\partial B_\perp$ is the (here, high-field) Hall coefficient, whose negative sign is consistent with electron-like transport. Making use of the Drude's formula, it follows for μ_H, the electronic mobility:

$$\mu_H = \frac{\sigma_s}{en_{2D}}, \tag{37}$$

where $\sigma_s = \sigma_{xx}$ is the sheet conductance of the considered Hall bar device (along $[\bar{1}10]$). Figure S6(c) displays both the estimated areal carrier density and electron mobility of the $[111]-LaAlO_3/SrTiO_3$ 2DES across the whole accessible doping range $0.5 \leq \sigma_{xx} \leq 6$ mS. While the mobility is found to increase monotonically, the apparent decrease of n_{2D} versus σ_{xx} is consistent with previous reports making use of a two-band fitting procedure up to 15 T, and which has been physically mapped by self-consistent tight-binding calculations to the redistribution of sub-bands population under the effect of electronic correlations (see Ref. 2 for details).

We estimate the momentum relaxation time $\tau_{(el)}$ [see Figure S6(d)], within Drude's model, which in the quasi-d.c. limit

($\omega\tau \ll 1$) is given by:

$$\tau = \frac{\mu_{\rm H} m^*}{e}, \tag{38}$$

where $m^* = \sqrt{m^*_{[\bar{1}\bar{1}2]} \cdot m^*_{[\bar{1}10]}}$ is the effective mass of the multi-orbital 2DES in the $SrTiO_3$[111] quantum well, with $m^*_{[\bar{1}\bar{1}2]} = 8.7 m_e$ and $m^*_{[\bar{1}10]} = 1.1 m_e$ [10], m_e the electron mass. The resulting calculated m^* is assumed to be gate- and temperature-independent.

A linear interpolation of the measured value of the momentum relaxation time τ vs. $\sigma_{\rm xx}$ allows to calculate the sheet conductance dependence of the BCD's magnitude $D_{\rm x}$ (shown in Fig. 4**d**) following equation (1) of the main manuscript.

Ordinary Hall effect & estimation of the momentum relaxation time − temperature dependence

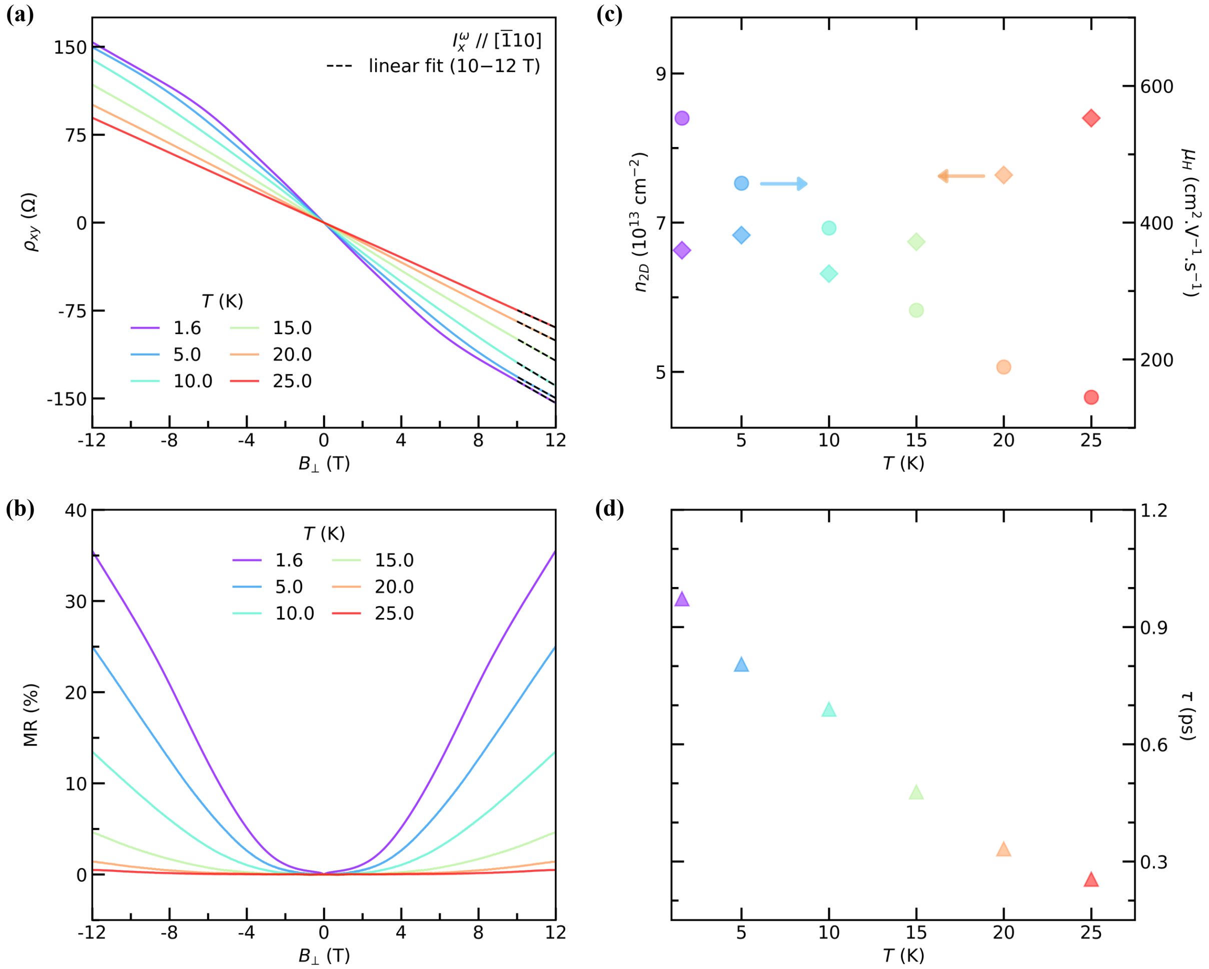

Figure S 7. **Temperature dependent ordinary Hall effect and magnetoresistance of the 2DES. (a)** Temperature dependent Hall effect response of the 2DES for the current sourced along [$\bar{1}$10]. Dashed black lines are linear fits to $\rho_{\rm xy}(B_\perp)$ performed between 10 and 12 T. **(b)** Corresponding longitudinal magnetoresistance. $B_\perp$, the out-of-plane magnetic field. **(c)** Experimentally estimated sheet carrier density $n_{\rm 2D}$ (left axis) and electronic mobility $\mu_{\rm H}$ (right axis) as a function of temperature. **(d)** Momentum relaxation time τ versus temperature T, obtained in a Drude model, following Eq. (38)

Additionally, we also report the temperature dependence of the ordinary Hall effect and longitudinal MR of the 2DES, as shown in Fig. S7(a) and S5(b), respectively, for the Hall bar device oriented along [$\bar{1}$10]. Following the steps described above for the gate-dependent data set, we similarly extract the temperature dependence of the carrier density and electronic mobility [see Fig. S7(c)], as well as that of the momentum scattering time [see Fig. S7(d)]. Even though not shown here, we perform the

same analysis for the orthogonal Hall bar device (*i.e.*, oriented along $[\bar{1}\bar{1}2]$) for experimentally measured data between 1.5 K and 30 K. These combined results, together with nonlinear Hall measurements with time-reversal symmetry, allow us to calculate the temperature dependence of the nonlinear conductivity tensor elements χ_{yxx} and χ_{xyy} [see Fig. 4**e** of the main manuscript], as well as the T-dependence of the Berry curvature dipole D_{x} [see Fig. 4**f** of the main manuscript].

Additional doping-dependent Hall effect measurements and magnetoconductance in the weak antilocalization regime

We present in Fig. S8**(a,b)** the gate-dependent ordinary Hall and longitudinal magnetoconductance data set collected for the Hall bar device oriented along the $[\bar{1}\bar{1}2]$ direction. This allows the experimental estimation of total the carrier density (n_{2D}), Hall mobility (μ_{H}), momentum scattering time (τ_{el}), as well as inelastic and spin-orbit and scattering times (τ_{i} and τ_{so}, respectively) as a function of the 2DES' sheet conductance σ_{yy} ($I^{\omega}_{\mathrm{y}} \parallel [\bar{1}\bar{1}2]$).

We refer the reader to the Methods sections of the main manuscript for details regarding the Hikami-Larkin-Nagaoka (HLN) model used to fit [see Eq. (3)] the magnetoconductance curves in the weak-antilocalization regime [11, 12].

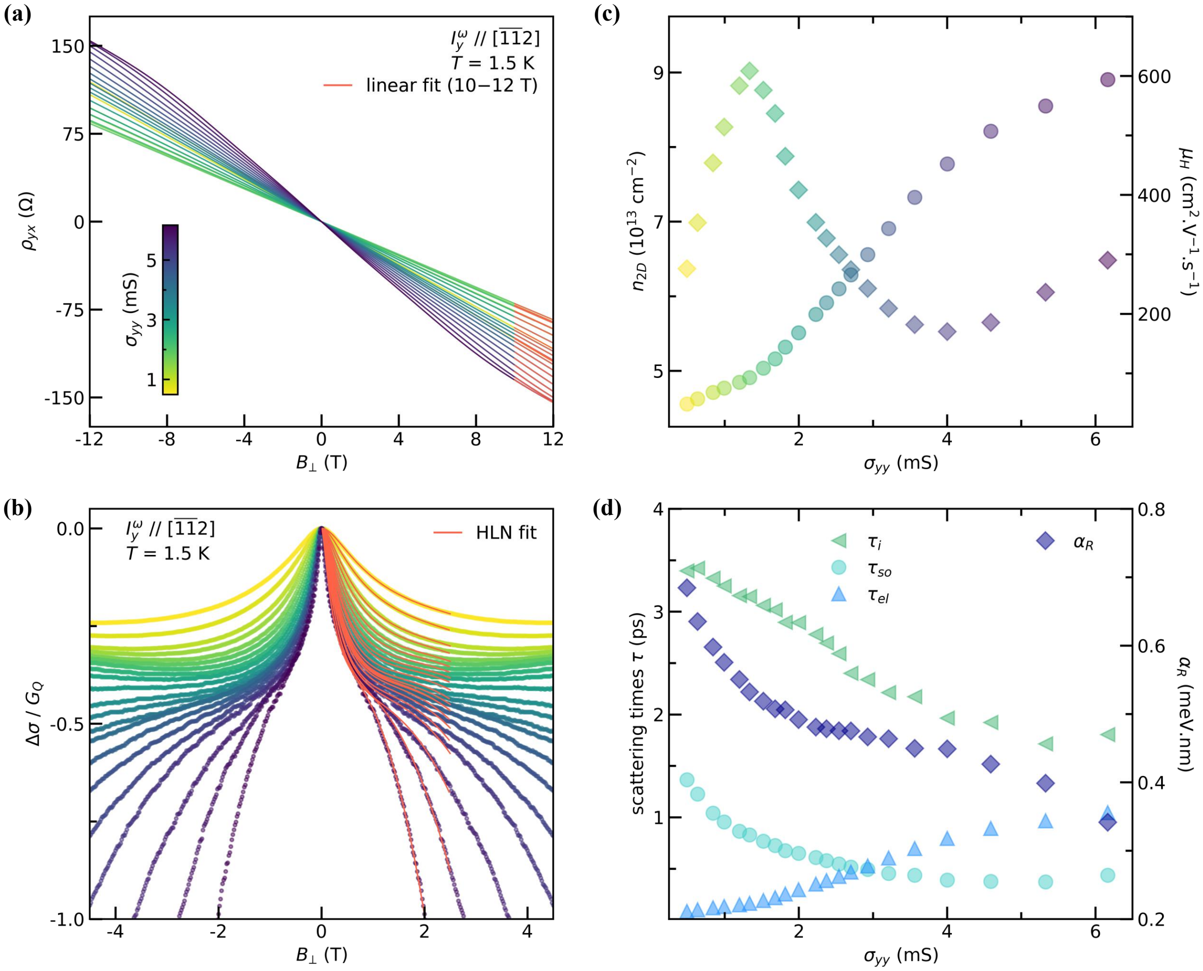

Figure S 8. **Ordinary Hall effect and magnetoconductance in the WAL regime.** **(a)** Gate-modulated ordinary Hall effect response of the 2DES for the current sourced along $[\bar{1}\bar{1}2]$. Solid red lines are linear fits to $\rho_{\mathrm{yx}}(B_{\perp})$ performed between 10 and 12 T. **(b)** Corresponding magnetoconductance curves (normalized to the quantum of conductance G_{Q}) and fitted (solid red lines)using a Hikami-Larkin-Nagaoka model, following Eq. (3) from the main manuscript. $B_{\perp}$, the out-of-plane magnetic field. **(c)** Experimentally estimated sheet carrier density n_{2D} (left axis) and electronic mobility μ_{H} (right axis) versus sheet conductance σ_{yy}. **(d)** Left axis: Momentum, inelastic and spin-orbit relaxation times τ_{el}, τ_{i} and τ_{so}, as a function of σ_{yy}. Right axis: Strength of the Rashba spin-orbit coupling α_{R} *vs.* σ_{yy}.

Estimation of the critical magnetic field and corresponding Zeeman energy. Comparison with the Rashba spin-orbit energy

On one hand, we establish a criterion for the determination of the 'critical' magnetic field, B^c, at which the transverse magnetoresponse in a planar magnetic field exceeds a given threshold value R^c_{xy} [shown in the legends of panels S9**(a,c)**, respectively]. The corresponding effective Zeeman energy, at the in-plane field magnitude $B = B^c$, is given by:

$$\Delta^c_Z = \Delta_Z(B^c) = \frac{1}{2} g \mu_B B^c \,, \tag{39}$$

where $g = 2$ is the electron g-factor. In the main manuscript, we keep the criterion $R^c_{xy} \equiv |R_{xy}| \geq 6 \;\; \Omega$ for the determination of B^c and Δ^c_Z, as shown in Extended Data Fig. 5.

On the other hand, assuming a D'yakonov-Perel' spin relaxation mechanism [13, 14], the Rashba spin-orbit energy is given by [12]:

$$\Delta_{so} = 2\alpha_R k_F \,, \tag{40}$$

with α_R the Rashba spin-orbit coupling (SOC) determined from WAL measurements (see Methods section), and k_F the electron wavevector at the Fermi energy.

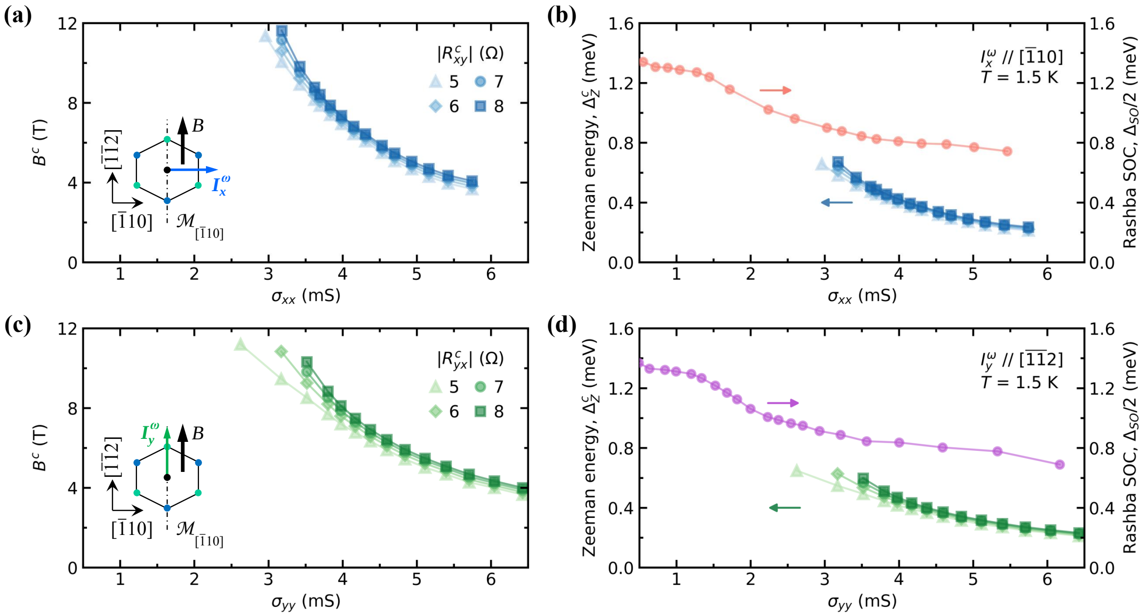

Figure S 9. **Estimation of the critical magnetic field, corresponding Zeeman energy and comparison with the Rashba spin-orbit energy.** **(a)** Estimation of the critical field B^c corresponding to the onset of the planar Hall effect, for I^ω_x along $[\bar{1}10]$. **(b)**, Corresponding effective Zeeman energy Δ^c_Z at $B = B^c$ (left axis), and Rashba spin-orbit energy $\Delta_{so}/2$ (right axis) versus sheet conductance. **(c-d)** *Idem* for I^ω_y along $[\bar{1}\bar{1}2]$.

Out-of-plane misalignment of the planar magnetic field

In order to experimentally estimate the value of the out-of-plane misalignment angle, γ, for the measurements displayed in Fig. 3**c,d** of the main manuscript, we conduct a low-field analysis of both the ordinary Hall and first-harmonic planar Hall effects as a function of doping levels for the same $[\bar{1}10]-$oriented Hall bar device.

Figures S10(a) and S8(b) are magnified low-field views of the data set displayed in Fig. S6(a) and Fig. 2**c** (see main manuscript). We perform linear fits between ± 2 T of the field-antisymmetrized out-of-plane $\rho_{xy}(B_\perp)$, and field-antisymmetrized in-plane $R^\omega_{xy}(B_\parallel)$ Hall magnetoresponses. The corresponding slopes are shown in Fig. S10(c) and S10(d), respectively. The common dependence of both quantities as a function of σ_{xx} highlights their common origin. We hence attribute the linear contribution to $R_{xy}(B)$ in Fig. 2**c** [here denoted: $R^\omega_{xy}(B_\parallel)$] at low-field to a spurious contribution of the ordinary Hall component due to a small

out-of-plane magnetic field component: $\Delta B_{\perp} = B_{\parallel}\sin(\gamma)$, resulting from an imperfect coplanar alignment of the field with the plane of the 2DES. We denote this out-of-plane misalignment angle γ. The contribution ΔR^{ω}_{xy} to the planar Hall effect, from the conventional Hall effect due to this misalignment, is then expected to take the form: $\Delta R^{\omega}_{xy} = \frac{\delta \rho_{\mathrm{xy}}}{\delta B_{\perp}} \cdot B_{\parallel}\sin(\gamma)$.

To further support this interpretation, we display in Fig. S11 the calculated quantity γ given by:

$$\gamma = \sin^{-1}\left(\frac{\delta R^{\omega}_{\mathrm{xy}}/\delta B_{\parallel}}{\delta \rho_{\mathrm{xy}}/\delta B_{\perp}}\right) \tag{41}$$

where the argument is the ratio of the low-field slopes from the in-plane and out-of-plane Hall effects. We indeed find that γ is independent of σ_{xx}, and consistently smaller than 1.5°. This misalignment amounts to an out-of-plane field component $\Delta B_{\perp} \leq 25$ mT at $B_{\parallel} = 1$ T.

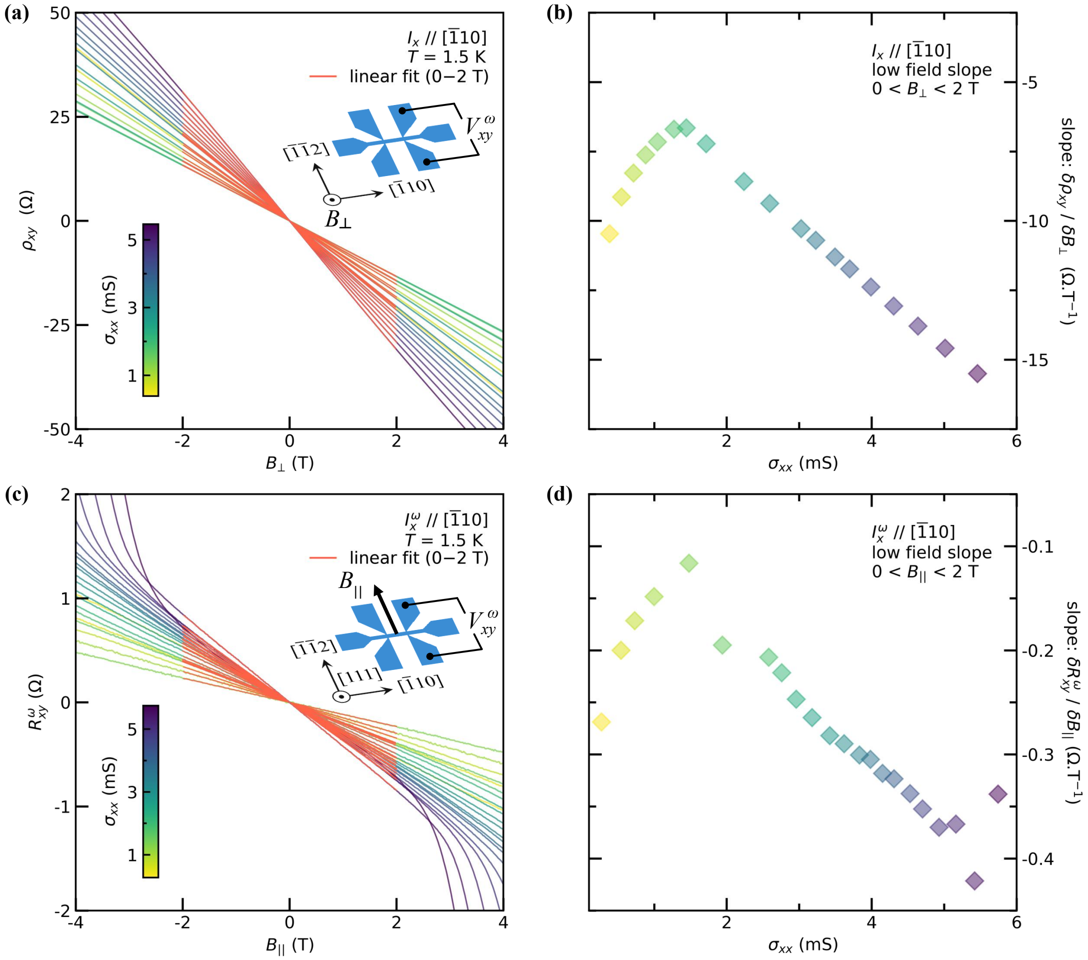

Figure S 10. **Low-magnetic field linear fitting of ordinary Hall effect and first Harmonic planar Hall effect. (a)** Gate-modulated ordinary Hall effect response vs. $B_{\perp}$ of the 2DES for I_{x} along $[\bar{1}10]$. **(b)** Planar Hall response versus $B_{\parallel}$, the out-of-plane magnetic field. Solid red lines are linear fits performed between 0 and 2 T. **(c),(d)** Corresponding low-field slopes of $\rho_{\mathrm{xy}}(B_{\perp})$, and $R^{\omega}_{\mathrm{xy}}(B_{\parallel})$, respectively.

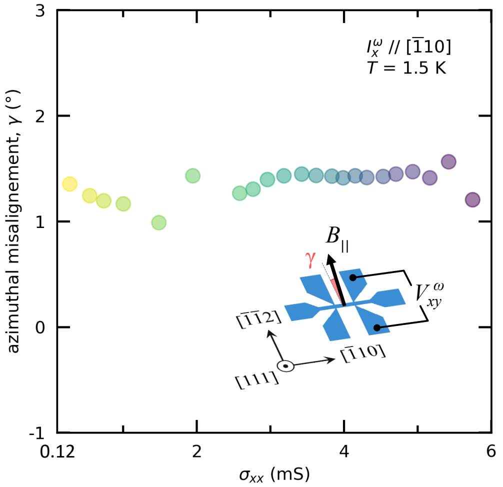

Figure S 11. **Out-of-plane misalignment angle.** Experimentally estimated out-of-plane tilt value γ of the planar magnetic field, following Eq. (41), for the measurement of R_{yx} displayed in Fig. 2**c** (see main manuscript).

Figure S12 displays the change of longitudinal and transverse planar magnetoresponses, when deliberately imposing a small out-of-plane misalignment of the magnetic field (with tilt angle $\Delta\gamma$). We define $\gamma = 0°$ the angle at which the measurements displayed in Fig. **2** of the manuscript were performed. Prior to any measurement campaign, we tentatively minimize γ by finding the tilt-angle which minimizes the low-field slope of $R^{\omega}_{\text{xy}}(B_{\parallel})$, which is typically performed at low doping levels where the BCD-induced planar Hall contribution is absent.

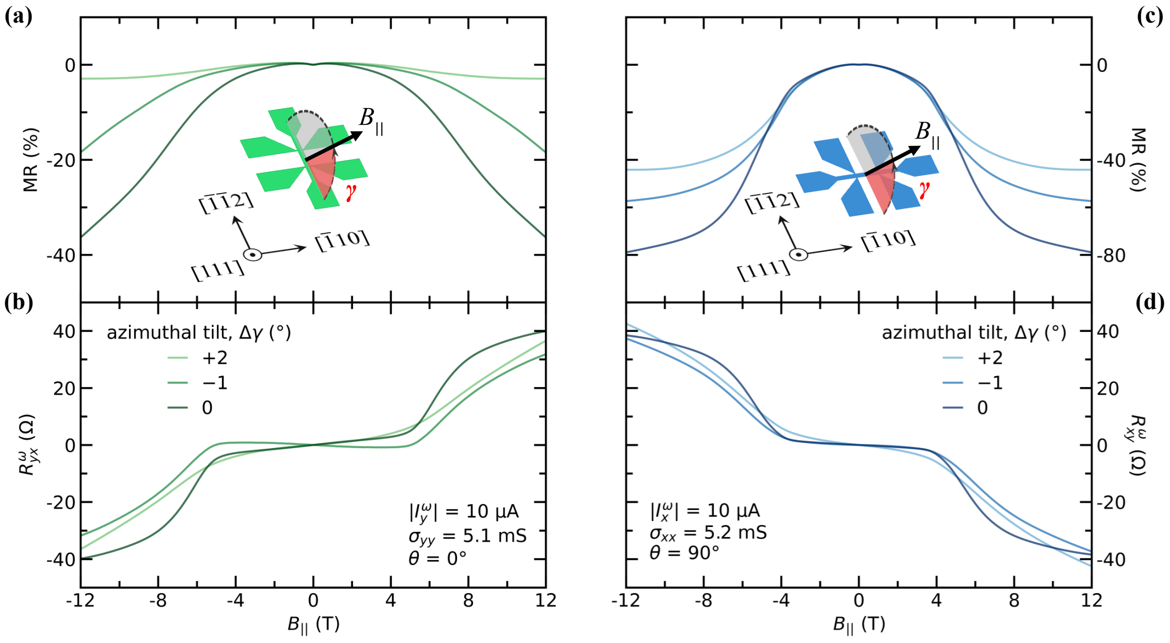

Figure S 12. **Dependence of planar MR and planar Hall signals in a noncoplanar magnetic field.** Dependence of **(a)** the longitudinal MR, and **(b)** linear Hall response of the $[\bar{1}\bar{1}2]$ device ($\approx // B$), for small out-of-plane deviations ($\Delta\gamma$) of the magnetic field. **(c)**,**(d)**, Idem with B perpendicular to I^{ω} sourced along $[\bar{1}10]$ device. For both devices: while the linear planar Hall response is found to be quite insensitive to small $\Delta\gamma$ offsets, the quasi-planar MR shows a drastic change of magnitude upon small tilts of the magnetic field out-of-plane.

As seen in Fig. S12(a,c), we find that the planar longitudinal MR is extremely sensitive to a very small out-of-plane misalignment, changing by a factor of two within only one degree. This explains the relative magnitude discrepancies in the planar MR between different cooldowns and measurements campaign. On the other hand, the planar Hall contribution is found to be relatively robust against small out-of-plane tilts of the magnetic field [see Fig. S12(b,d)]. The low-field slope R_{yx} is found to be

proportional to $\Delta\gamma$, corroborating that it originates from a spurious conventional Hall component.

Angular dependence of planar magnetoresponses

We acquire the full in-plane angular dependence of the longitudinal magnetoresistance (MR) at $|B| = 12$ T, by sweeping the angle θ between the field and the current direction, in steps of $5°$. Due to our mechanical rotator being limited to a $180°$ rotation range, we perform the rotation procedure twice, once for $B = +12$ T and a second time for $B = -12$ T, while keeping the sourced current and voltage probe contacts polarities unchanged.

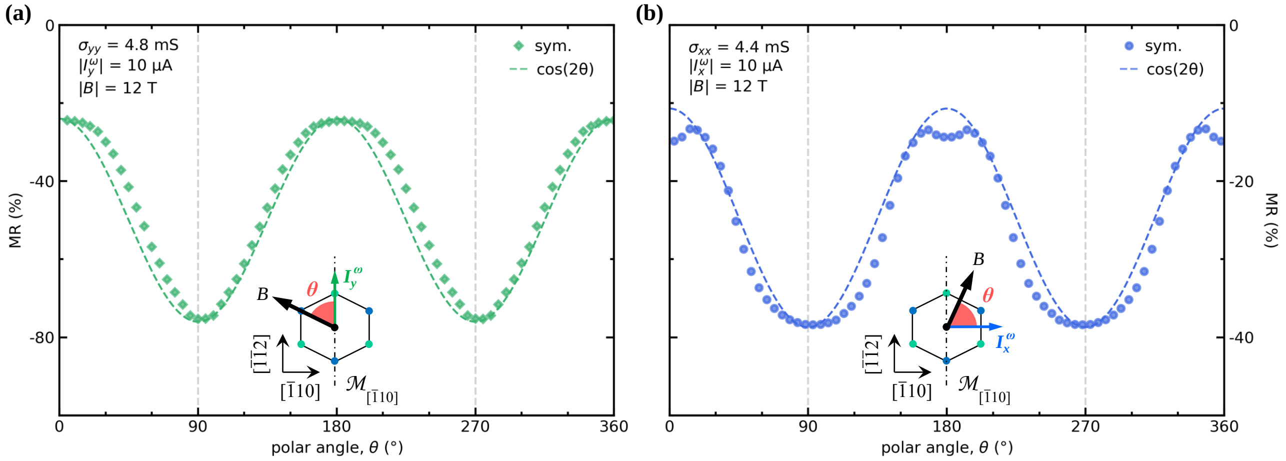

Figure S 13. **Angular dependence of planar magnetoresistance.** Dependence of the field-symmetric planar magnetoresistance upon rotating a 12 T magnetic field within the sample's plane for **(a)** I_y^ω along $[\bar{1}\bar{1}2]$, and **(b)** I_x^ω along $[\bar{1}10]$; at a fixed sheet conductance value. θ is defined as the angle between the current bias direction and the external magnetic field. Dotted lines display the $\cos(2\theta)$ dependence of the MR.

We can obtain the full field-symmetrized magnetoresistance at $|B| = 12$ T, as displayed in Fig. S13, by virtue of Onsager's relation: $\rho_{\text{xx,yy}}(B) = \rho_{\text{xx,yy}}(-B)$. The field-symmetric longitudinal magnetoresistance is then given by:

$$\text{MR} = \frac{\rho_{\alpha\alpha}(B) + \rho_{\alpha\alpha}(-B)}{2\rho_{\alpha\alpha}(0)} - 1. \tag{42}$$

Whether the bias current is sourced along $\hat{\boldsymbol{y}} \parallel [\bar{1}\bar{1}2]$ or along $\hat{\boldsymbol{x}} \parallel [\bar{1}10]$, the planar MR follows the semiclassical $\cos(2\theta)$ dependence, where θ is the relative angle between the current direction (along a principal crystal axis) and the planar magnetic field orientation.

Concomitantly, when measuring the planar transverse magnetoresponse, we observe that the field-symmetric contributions $R^{\text{sym}}_{xy(yx)}$, of semiclassical origin (usually referred to as the 'planar Hall effect'), follows a $\sin(2\theta)$ dependence, and goes to zero at $\theta = 0[\frac{\pi}{2}]$, as expected for a nonmagnetic system.

However, we find that the total transverse resistance $R^{\text{tot}}_{\alpha\beta}$ (displayed in Fig. 2**f** of the manuscript) is dominated by the field-antisymmetric contribution (see Fig. S14) , $R^{\text{as}}_{\alpha\beta}$, dubbed "anomalous planar Hall effect" [4, 15], which remains finite whenever the external planar magnetic field is not orthogonal to the mirror line $\mathscr{M}_{[\bar{1}10]}$. Independent of whether the current is sourced along the $[\bar{1}10]$ or $[\bar{1}\bar{1}2]$ crystal axis directions, the transverse planar contribution vanishes in the linear response regime when the ($\mathscr{M}_{[\bar{1}10]}$-symmetry preserving) planar magnetic field is aligned with the $[\bar{1}10]$ direction (*i.e.* $\varphi = 0[\pi]$).

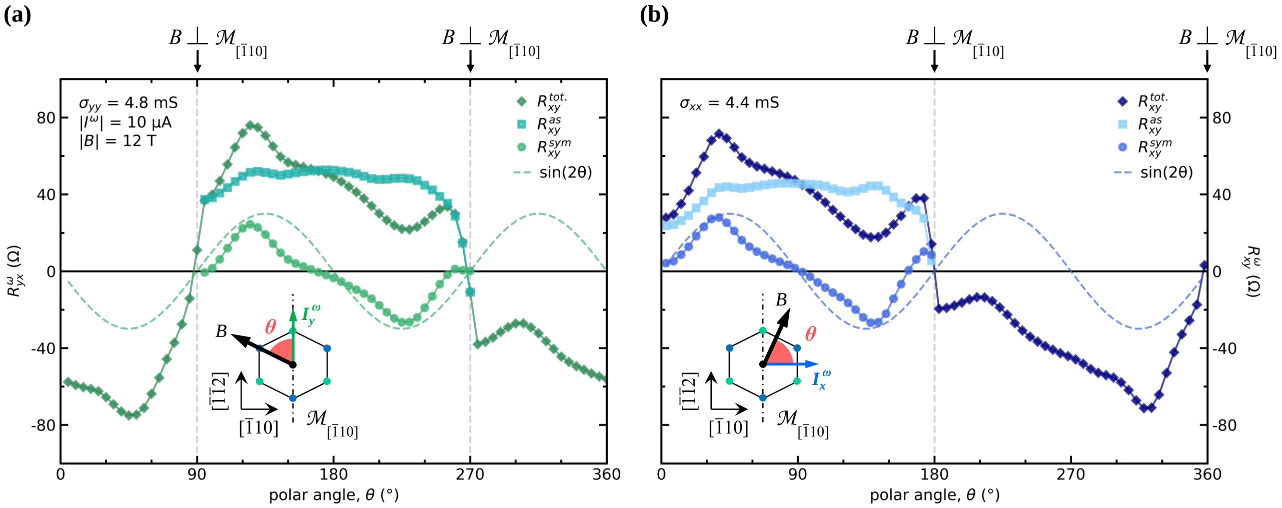

Figure S 14. **Angular dependence of transverse magnetoresistance contributions.** Measured raw total signal $R_{\alpha\beta}^{\text{tot.}}$ at $|B| = 12$ T and the corresponding field-antisymmetric $R_{\alpha\beta}^{\text{as}}$ and field-symmetric $R_{\alpha\beta}^{\text{sym}}$ contributions for **(a)** I_y^{ω} along $[\bar{1}\bar{1}2]$, and **(b)** I_x^{ω} along $[\bar{1}10]$ at fixed electronic density. Remarkably, for both devices orientations, the semiclassical contribution to the planar Hall effect, *i.e.*, $R_{\text{yx(xy)}}^{sym}$, follows the expected $\sin(2\theta)$ dependence (highlighted by the dotted lines).

Supplementary References

[1] R. Barnett, G. R. Boyd, and V. Galitski, Phys. Rev. Lett. **109**, 235308 (2012).
[2] A. M. R. V. L. Monteiro, M. Vivek, D. J. Groenendijk, P. Bruneel, I. Leermakers, U. Zeitler, M. Gabay, and A. D. Caviglia, Phys. Rev. B **99**, 201102 (2019).
[3] G. M. De Luca, R. Di Capua, E. Di Gennaro, A. Sambri, F. M. Granozio, G. Ghiringhelli, D. Betto, C. Piamonteze, N. B. Brookes, and M. Salluzzo, Phys. Rev. B **98**, 115143 (2018).
[4] R. Battilomo, N. Scopigno, and C. Ortix, Phys. Rev. Research **3**, L012006 (2021).
[5] N. Wadehra, R. Tomar, R. M. Varma, R. K. Gopal, Y. Singh, S. Dattagupta, and S. Chakraverty, Nature Communications **11**, 874 (2020).
[6] P. K. Rout, E. Maniv, and Y. Dagan, Phys. Rev. Lett. **119**, 237002 (2017).
[7] J. Biscaras, N. Bergeal, S. Hurand, C. Grossetête, A. Rastogi, R. C. Budhani, D. LeBoeuf, C. Proust, and J. Lesueur, Phys. Rev. Lett. **108**, 247004 (2012).
[8] A. Joshua, S. Pecker, J. Ruhman, E. Altman, and S. Ilani, Nature Communications **3**, 1129 (2012).
[9] U. Khanna, P. K. Rout, M. Mograbi, G. Tuvia, I. Leermakers, U. Zeitler, Y. Dagan, and M. Goldstein, Phys. Rev. Lett. **123**, 036805 (2019).
[10] T. C. Rödel, C. Bareille, F. Fortuna, C. Baumier, F. Bertran, P. Le Fèvre, M. Gabay, O. Hijano Cubelos, M. J. Rozenberg, T. Maroutian, P. Lecoeur, and A. F. Santander-Syro, Phys. Rev. Applied **1**, 051002 (2014).
[11] S. Hikami, A. I. Larkin, and Y. Nagaoka, Progress of Theoretical Physics **63**, 707 (1980).
[12] S. Maekawa and H. Fukuyama, Journal of the Physical Society of Japan **50**, 2516 (1981).
[13] M. I. D'Yakonov and V. I. Perel', Soviet Journal of Experimental and Theoretical Physics Letters **13**, 467 (1971).
[14] M. Dyakonov and V. Perel, Physics Letters A **35**, 459 (1971).
[15] J. H. Cullen, P. Bhalla, E. Marcellina, A. R. Hamilton, and D. Culcer, Phys. Rev. Lett. **126**, 256601 (2021).
